# Clarifying the Dopant Local Structure and Effect on Ionic Conductivity in Garnet Solid-State Electrolytes for Lithium-Ion Batteries

**DOI:** 10.1021/acs.chemmater.3c01831

**Published:** 2023-11-14

**Authors:** Sundeep Vema, Astrid H. Berge, Supreeth Nagendran, Clare P. Grey

**Affiliations:** † Yusuf Hamied Department of Chemistry, University of Cambridge, Lensfield Road, Cambridge CB2 1EW, U.K.; ‡ The Faraday Institution, Quad One, Harwell Campus, Didcot OX11 0RA, U.K.

**Keywords:** solid electrolyte, LLZO, dopant site, nuclear magnetic resonance spectroscopy, side-product, ionic conductivity, lithium excess, tetragonal phase

## Abstract

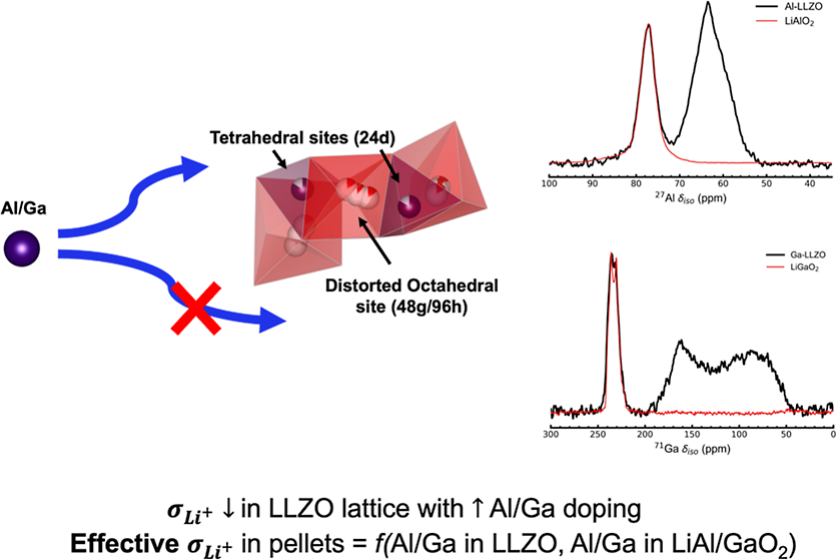

The high Li-ion conductivity and wide electrochemical stability of Li-rich garnets (Li_7_La_3_Zr_2_O_12_) make them one of the leading solid electrolyte candidates for solid-state batteries. Dopants such as Al and Ga are typically used to enable stabilization of the high Li^+^ ion-conductive cubic phase at room temperature. Although numerous studies exist that have characterized the electrochemical properties, structure, and lithium diffusion in Al- and Ga-LLZO, the local structure and site occupancy of dopants in these compounds are not well understood. Two broad ^27^Al or ^69,71^Ga resonances are often observed with chemical shifts consistent with tetrahedrally coordinated Al/Ga in the magic angle spinning nuclear magnetic resonance (MAS NMR) spectra of both Al- and Ga-LLZO, which have been assigned to either Al and/or Ga occupying 24d and 96h/48g sites in the LLZO lattice or the different Al/Ga configurations that arise from different arrangements of Li around these dopants. In this work, we unambiguously show that the side products γ-LiAlO_2_ and LiGaO_2_ lead to the high frequency resonances observed by NMR spectroscopy and that both Al and Ga only occupy the 24d site in the LLZO lattice. Furthermore, it was observed that the excess Li often used during synthesis leads to the formation of these side products by consuming the Al/Ga dopants. In addition, the consumption of Al/Ga dopants leads to the tetragonal phase formation commonly observed in the literature, even after careful mixing of precursors. The side-products can exist even after sintering, thereby controlling the Al/Ga content in the LLZO lattice and substantially influencing the lithium-ion conductivity in LLZO, as measured here by electrochemical impedance spectroscopy.

## 1 Introduction

Solid-state batteries have been projected to enable energy storage devices with higher energy density and thermal stability than current, state-of-the-art organic liquid electrolyte-based batteries.^1,2^ A solid electrolyte is the key component of a solid-state battery that enables these features, and consequently, a wide range of lithium-containing inorganic oxides, sulfides, and nitrides have been explored as solid electrolytes. Li-rich garnets (Li_7_La_3_Zr_2_O_12_, LLZO) possess high room temperature (RT) ionic conductivity and a relatively wide electrochemical stability as compared to other solid electrolytes, and have therefore been a subject of intense interest in the past few years.^3^

LLZO with cubic *Ia*3®*d* symmetry was synthesized at 1230 °C by Murugan et al.,^4^ for the first time in 2007 and was shown to have high RT ionic conductivity (∼3 × 10^–4^ S cm^–1^) with a low activation energy of 0.3 eV. However, Awaka et al.,^5^ reported that LLZO synthesized at 900 °C crystallizes in a tetragonal structure, *I*4_1_/*acd*, through single crystal and powder X-ray diffraction (XRD) and neutron diffraction. A relatively low ionic conductivity (∼10^–6^ S cm^–1^) with a high activation energy (0.54 eV) was also reported. A follow-up study on single crystals of LLZO synthesized at 1250 °C noted the formation of cubic LLZO, where Li occupied a combination of tetrahedral sites (24d) and distorted octahedral sites (96h).^6^ In a high-temperature XRD study performed on Li_7_La_3_Sn_2_O_12_, a compound with a similar structure to LLZO, Percival et al.,^7^ observed the transformation from a tetragonal to a cubic unit cell above 750 °C, but the compound transformed back to tetragonal on cooling to RT. It was hypothesized that LLZO synthesized at high temperatures (above 1200 °C) might result in disordered structures that could be stabilized by Li loss during cooling to form cubic Li_7–*x*_La_3_Zr_2_O_12–*x*/2_.

Through a systematic study of LLZO synthesis at 900–1100 °C in two different crucibles, Al_2_O_3_ and platinum, Geiger et al.^8^ revealed the origin of cubic LLZO. They showed that a small amount of aluminum from the Al_2_O_3_ crucible was incorporated into the LLZO lattice, leading to the transformation from a tetragonal to cubic structure. Consistent with this, only the tetragonal phase was seen at room temperature in LLZO synthesized in platinum crucibles. These observations were supported by using a range of techniques, including electron probe microanalysis, laser ablation inductively coupled plasma mass spectrometry, and ^27^Al MAS NMR spectroscopy. The aluminum-induced cubic transformation of LLZO was subsequently confirmed by intentional addition of Al_2_O_3_ during synthesis in later studies.^9−12^

These observations suggested that Li content reduction in the LLZO framework through multivalent dopants could lead to the stabilization of the highly conductive cubic lattice and thus, different combinations of dopants (Al, Ga, Fe, Ta, W, Nb, etc.) have been explored to stabilize the cubic polymorph of LLZO and to obtain high Li-ion conductivities.^13−18^ Among various doped LLZO compounds reported in the literature, Al doped LLZO (Al-LLZO) and Ga doped LLZO (Ga-LLZO) have high RT ionic conductivity (∼10^–4^ and 10^–3^ S cm^–1^, respectively), making them promising candidates for commercial applications. Although numerous studies exist that have characterized the electrochemical properties,^19,20^ structure,^19,21,22^ and nature of lithium diffusion in Al- and Ga-LLZO,^23−31^ the nature of the local structure, and the site occupancy of the dopants is still unclear.

**Figure 1:**
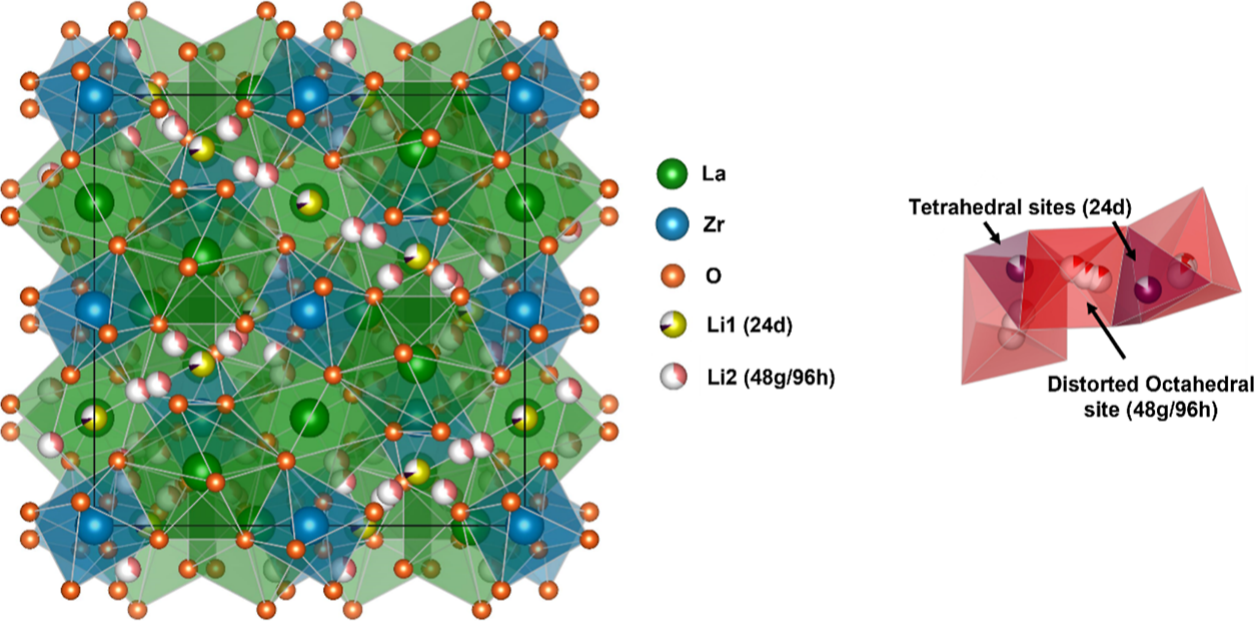
Crystal structure of cubic Al-LLZO (space group *Ia*3®*d*) (left). The structure has been generated with VESTA 3.4.7.^32^ Representation of the interconnected Li network in Al-LLZO (right), where the Li1 tetrahedral sites (24d) and the Li2 distorted octahedral sites (48g/96h) are connected by face-sharing tetrahedra/octahedra. Both these Li sites have been reported as possible sites for Al/Ga.

One of the earliest studies investigating the local structure of Al in Al-LLZO was performed by Geiger et al.,^8^ who reported that Al occupies two distinct sites in LLZO based on the two different signals observed in the tetrahedral region of the ^27^Al MAS NMR spectrum of Al-LLZO powder collected at 9.4 T. The signal at ∼68 ppm was assigned to Al in a tetrahedral site (24d), and the signal at ∼81 ppm to a distorted octahedral site (96h), which can also be viewed as a distorted 5-fold coordination environment (Figure 1, left). Buschmann et al.^33^ and Kuhn et al.^34^ assigned the higher shifted resonance in the ^27^Al MAS NMR spectrum to Al^3+^ in tetrahedral sites adjacent to La^3+^ or Zr^4+^ vacancies. In a different study by Düvel et al.,^35^ a series of Al-LLZO compounds were prepared with increasing Al^3+^ contents via high-energy mechanical milling followed by annealing at 600 °C, and the ^27^Al MAS NMR spectra of the compounds were measured. Multiple resonances were observed in addition to the two resonances discussed above, which were attributed to Al^3+^ occupation of either La^3+^ or Zr^4+^ sites in the LLZO lattice. Rettenwander et al.,^36^ performed DFT calculations to determine the origin of the two resonances seen in ^27^Al MAS NMR spectra and proposed that Al^3+^ occupied both tetrahedral (24d) and 4-fold coordinated distorted octahedral (96h) sites based on the similar calculated energies for Al^3+^ occupation of the sites. Small displacements of Al^3+^ ions were observed to lead to a distribution of different local oxygen coordination environments, and this was hypothesized as a reason for the existence of broad resonances in the ^27^Al MAS NMR spectra. In a further high-field MAS NMR study performed on Al_*x*_Ga_*y*_Li_7–3(*x*+*y*)_La_3_Zr_2_O_12_ solid solutions by Rettenwander et al.,^37^ the existence of two Al^3+^ environments in Al-LLZO powder was confirmed, and these were attributed to the two sites in the LLZO lattice as reported earlier. Similar observations have been made by high-resolution synchrotron XRD (SXRD), neutron powder diffraction, and Raman spectroscopy performed on Al-LLZO powder.^38−40^ In a recent study by some of us, through a combination of DFT and MAS NMR experiments, Karasulu and Emge et al.,^41^ argued that Al^3+^ occupancy of distorted octahedral site (96h) was energetically unfavorable and proposed that differences in the number of Li ions adjacent (i.e., in the first cation coordination shell) to the Al^3+^ dopant could lead to a series of different configurations, accounting for the different resonances seen in ^27^Al MAS NMR spectrum of Al-LLZO. Similar discussions concerning the occupancies of Ga^3+^ in the different sites in LLZO are also present in the literature.^41−45^

In this work, a systematic investigation of the local structure of the Al dopant in Al-LLZO and Ga dopant in Ga-LLZO with ^27^Al and ^71^Ga MAS NMR spectroscopy, respectively, was conducted on samples synthesized with differing amounts of Li excess in the precursors and on pellets sintered to produce particles with different grain sizes. Three different ^27^Al resonances, corresponding to two tetrahedral and one octahedral Al environment, were observed in the ^27^Al MAS NMR spectrum of Al-LLZO and two different ^71^Ga resonances, corresponding to two tetrahedral environments, were observed in the ^71^Ga MAS NMR spectrum of Ga-LLZO, as observed in earlier studies. Through MAS NMR performed on model compounds and by double quantum–single quantum (DQ–SQ) correlation experiments,^46^ which can be used to select for ^27^Al nuclei that are in close spatial proximity in the lattice, as found in the Al-containing side-products γ-LiAlO_2_ and LaAlO_3_, it was confirmed that the dopants occupy only one crystallographic site in both Al- and Ga-LLZO, and all other additional resonances were due to the side-products. It was further found that a significant amount of the element added as an LLZO-dopant can exist in side-products depending on the synthesis and sintering route. Electrochemical impedance spectroscopy (EIS) performed on different sintered samples showed that the dopant distribution between the side-products and LLZO lattice substantially influences the lithium-ion conductivity in LLZO solid electrolytes.

## 2 Experimental Methods

### 2.1 Synthesis of Al-LLZO and Ga-LLZO Powders

Al-LLZO and Ga-LLZO were synthesized using a solid-state method with excess Li (both for Al- and Ga-LLZO), and stoichiometric Li precursors (only for Al-LLZO) in MgO crucibles to avoid unintentional Al doping as described in detail in a previous study.^47^ The synthesized doped LLZO powders were transferred above 200 °C to a glovebox to prevent any reaction with moisture and stored in airtight vials.

For preparing hot-pressed samples, Al-LLZO and Ga-LLZO powders were first ground and sieved, and then hot-pressed at 1085 °C for 70–90 min using a custom-built induction coil-based uniaxial hot-press, as described in detail in a previous study.^47^ The hot-pressed cylinder was cut using a diamond disc, and the pellets were hand-polished inside a glovebox. The pellets were then ground using an agate mortar and pestle and stored in glovebox. To achieve pellets with large grain sizes, hot-pressed Al-LLZO pellets were sintered under flowing oxygen at 1200 °C for 18 h. The pellets were centered on a flat MgO crucible cap and surrounded by a bed of synthesized Al-LLZO powder to reduce decomposition of LLZO due to Li loss during sintering.

### 2.2 Synthesis of γ-LiAlO_2_, LiGaO_2_ and LiAl_0.5_Ga_0.5_O_2_ Powders

Al_2_O_3_ (TEM < 50 nm, Sigma-Aldrich) and Ga_2_O_3_ (99.999%, Alfa Aesar) were dried at 900 °C for 12 h and transferred above 250 °C to a desiccator to allow them to cool down to RT. Li_2_CO_3_ (99.997%, Alfa Aesar) was dried at 150 °C for 12 h. Precursors corresponding to 2 g of γ-LiAlO_2_, LiGaO_2_, and LiAl_0.5_Ga_0.5_O_2_ were stoichiometrically weighed and mixed for 20 min in a mortar and pestle with acetone solvent to ensure homogeneous mixing. The solvent was then evaporated, and the dried powder was calcined at 1000 °C for 6 h under O_2_ flow, ∼30 mL/min with a 5 °C/min heating rate in a tube furnace (Carbolite) followed by natural cooling. Al_2_O_3_ crucibles (SRX61, Almath crucibles) were used for the synthesis of γ-LiAlO_2_, whereas MgO crucibles (SRX61MGO, Almath crucibles) were used for the synthesis of LiGaO_2_ and LiAl_0.5_Ga_0.5_O_2_ to prevent any unintentional incorporation of aluminum into the powder. The synthesized powders were transferred around 100 °C to a desiccator and stored in airtight vials for further analysis.

### 2.3 X-ray Diffraction

The phase purity was confirmed by powder SXRD. Al-LLZO, γ-LiAlO_2_, LiGaO_2_, and LiAl_0.5_Ga_0.5_O_2_ powders were finely ground in a mortar and pestle, filled in capillaries, and sealed using epoxy. Al-LLZO and Ga-LLZO samples were ground and packed inside the glovebox to prevent any reaction with moisture in the air. The capillaries were then transported to the I11 beamline at the Diamond Light Source, Oxford, United Kingdom, and SXRD patterns were collected at RT in transmission mode (λ = 0.824978 or 0.49381 Å). The transmitted X-rays were detected by position-sensitive detectors. The SXRD patterns were then analyzed using FullProf Suite.^48^

### 2.4 Magic Angle Spinning Nuclear Magnetic Resonance Spectroscopy

All samples were ground in agate mortars and packed into 1.3 or 4 mm ZrO_2_ rotors. Al-LLZO and Ga-LLZO samples were packed inside the glovebox and the other samples (γ-LiAlO_2_ and LiGaO_2_) were packed outside in ambient atmosphere. ^27^Al and ^71^Ga MAS NMR spectra were acquired on 700 MHz (16.4 T) magnets with Avance III consoles using a Bruker 1.3 mm HXY probe. The MAS NMR experiments were performed at sample spinning speeds of 40 kHz for 1.3 mm rotors. One-pulse pulse programs with a small flip angle (∼π/24 and π/4 for ^27^Al and ^71^Ga, respectively) were used to collect the MAS NMR spectra. ∼π/24 flip angle was used in the case of ^27^Al for quantitative MAS NMR measurements. Due to the low abundance of the ^71^Ga nuclei, and hence low signal-to-noise ratio, a small flip angle, and hence quantitative measurements could not be done for Ga-LLZO. Instead, a π/4 flip angle was used to maximize the signal intensity. The spectra were then externally referenced against AlF_3_ powder (−17 ppm) for ^27^Al and Ga(NO_3_)_3_ powder dissolved in distilled water (0 ppm) for ^71^Ga. These reference compounds were also used for pulse length optimization.

Two-dimensional (2D)—double quantum-single quantum (DQ–SQ) experiments were performed on a 1 GHz (23.5 T) magnet with Avance NEO consoles using a Bruker 1.9 mm HX probe. These experiments were performed with samples in 1.9 mm rotors at a MAS speed of 42 kHz. The pulse optimization was done on a 1:1 mixture of Al_2_O_3_ and γ-LiAlO_2_. The DQ–SQ experiments were performed using the BR2_2_^1^ homonuclear recoupling sequence.^46^ The pulses were optimized to be central transition selective.

The MAS NMR spectra were processed and deconvoluted with Bruker Topspin 4.0.8 and dmfit software packages.^49^ The ^27^Al and ^71^Ga MAS NMR spectra were fitted with the Q-MAS 1/2 model to fit the central transition, assuming infinitely fast MAS, to obtain values of the quadrupolar coupling (*C*_Q_) and asymmetry parameter (η_Q_).

### 2.5 Scanning Electron Microscopy

A Tescan MIRA3 FEG-SEM was used to collect the SEM images with a 5 keV accelerating voltage and a 6–8 mm working distance. The samples were sputter-coated with platinum to reduce the charging effects during imaging. Energy-dispersive X-ray spectroscopy (EDS) was performed on an Oxford Instruments X-maxN 80 EDS system. EDS was performed using an electron beam with a 30 keV acceleration voltage and a 15 mm working distance.

### 2.6 Impedance Measurements

The polished pellets inside the glovebox were placed in a MgO crucible and transferred to a custom-made quartz tube and thermally etched in a furnace (Carbolite) with a custom-made gas setup to switch between argon and oxygen to remove the surface passivation layers.^47^ The pellets were heated under O_2_ at 500 °C for 1 h at the rate of 10 °C/min, and then cooled to 200 °C at 10 °C/min. The quartz tube was then purged with argon for 20 min and then transferred at 200 °C into the glovebox prechamber. For blocking electrode measurements, the thermally etched pellets were centered under a stainless-steel disc having a 5 mm Ø hole, and sputter-coated with gold on both sides of the pellets. Then the pellets were closed in Swagelok cells for further characterization. The impedance measurements were performed with an amplitude of 10 mV at frequencies from 7 MHz to 1 Hz using VSP-300 (Biologic). The impedance data was fit with an equivalent circuit using a custom-written code in Python to extract the bulk and grain boundary ionic conductivities.

## 3 Results and Discussion

### 3.1 ^27^Al MAS NMR of Al-LLZO and γ-LiAlO_2_

**Figure 2:**
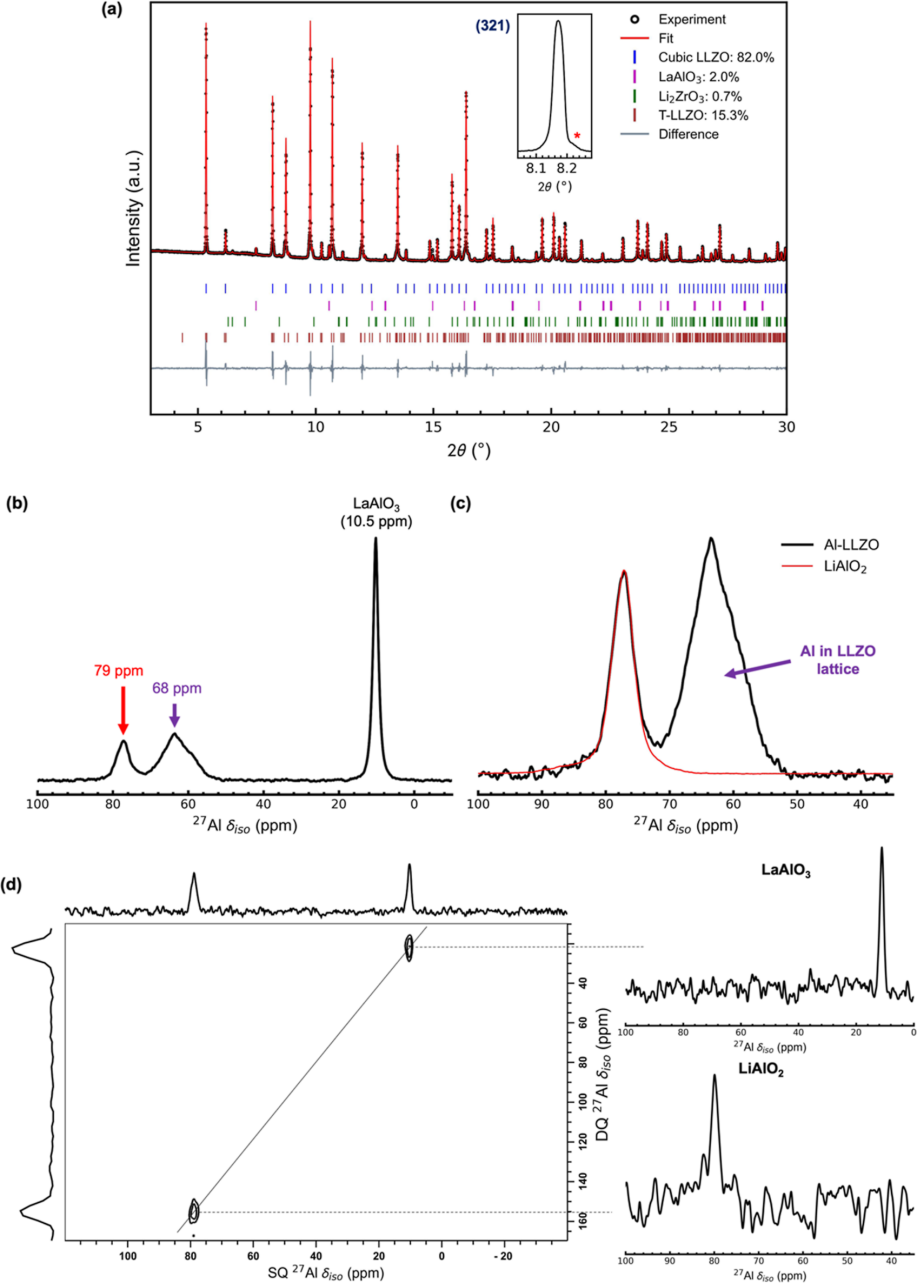
(a) SXRD pattern (λ = 0.493 Å) of Al-LLZO synthesized with 10% excess Li in the precursors along with its Rietveld refinement to extract phase fraction of LLZO and the side-products, the inset showing the (321) LLZO reflection (where * indicates the tetragonal LLZO phase). (b) ^27^Al MAS NMR spectrum of the same Al-LLZO sample; the isotropic chemical shifts (in ppm) of the three resonances are marked. (c) An enlargement of the same spectrum to show the resonances corresponding to Al in tetrahedral environments (black). The LLZO ^27^Al spectrum is overlaid with the spectrum of γ-LiAlO_2_ (red curve), where the γ-LiAlO_2_ spectrum was scaled to match the intensity of the resonance at 79 ppm in the Al-LLZO spectrum. (d) DQ–SQ 2D spectrum for the same Al-LLZO sample with a refocusing time of 6 rotor periods, performed here to probe the spatial proximity between ^27^Al nuclei; slices through the indirect (DQ) dimension are shown on the right, taken at positions indicated by the dashed lines. Where appropriate, the assignments of the resonances are marked (see text).

Al-LLZO (Al_0.36_Li_5.92_La_3_Zr_2_O_12_) was synthesized first with 10% extra Li in the precursors (referred to as Al-LLZO + 10% Li), excess Li_2_CO_3_, being used here to account for Li loss at high temperatures. The resulting high-resolution SXRD pattern is shown in Figure 2a. Rietveld refinement showed that the Al-LLZO + 10% Li sample was composed of cubic Al-LLZO (∼82%), tetragonal (Al)-LLZO (∼15%), and side-products LaAlO_3_ (∼2%) and Li_2_ZrO_3_ (∼0.7%). Its ^27^Al MAS NMR spectrum showed three distinct resonances (Figure 2b). Since Al is a quadrupolar nucleus, the spectrum was simulated to account for the second-order quadrupolar-induced shifts, and values for the isotropic chemical shifts of the resonances of ∼79, ∼68, and ∼10.5 ppm were extracted (Figure S1). These values are similar to those reported in the literature.^8,33,34,37,41^ Al in tetrahedral environments (AlO_4_) has been reported to have chemical shifts of around 50 to 90 ppm, whereas Al in octahedral environments (AlO_6_) has shifts of around −10 to 20 ppm.^50^ Thus, the first two resonances can tentatively be ascribed to Al in a tetrahedral environment, and the third resonance to Al in an octahedral environment. The signal at ∼10.5 ppm is therefore ascribed to the side-product, LaAlO_3_, which was also observed in the SXRD pattern.

While the use of excess Li in the synthesis of Li containing compounds is common, it can also lead to the formation of Li-containing side-products, whose concentration and nature can also vary depending on the chemical composition of the precursor. Gamma LiAlO_2_ (γ-LiAlO_2_) has been observed as a side-product in XRD analysis of Al rich Al-LLZO powders in a few studies,^51−53^ and hence it was synthesized in this study, and its SXRD pattern is shown in Figure S2. Its ^27^Al MAS NMR spectrum was collected, and an excellent match between the γ-LiAlO_2_ and the resonance in the LLZO sample with an isotropic shift of ∼79 ppm is seen on overlaying the two spectra (Figure 2c).

To further confirm the existence of γ-LiAlO_2_ in the LLZO sample, an ^27^Al DQ–SQ MAS NMR experiment was performed. In this experiment, only ^27^Al nuclei that are in close proximity to other ^27^Al nuclei are selected by the pulse sequence. Thus, only signals from Al-rich phases will be detected. The experiment makes use of the ^27^Al–^27^Al dipolar coupling, the dipolar coupling depending on the distance between ^27^Al nuclei (to the inverse third power). In practice, the dipolar coupling is reintroduced during the so-called mixing time or dephasing time of this two-dimensional (2D) experiment, longer mixing times connecting more distant nuclei/atoms. A signal on the “diagonal”, namely at a frequency 2ν in the indirect (first), and ν in the direct (second) dimension in the 2D DQ–SQ spectrum indicates that two or more Al nuclei with identical isotropic shifts are present in close proximity.^46,54^

Experiments with a model compound comprising 50% γ-LiAlO_2_ + 50% γ-Al_2_O_3_ were first performed, γ-LiAlO_2_ containing tetrahedrally coordinated Al^3+^ ions nearby four Al^3+^ ions in their first cation coordination shells at around 3.1 Å.^55^ Signal were seen at ∼79 and ∼15 ppm in the SQ dimension as expected for γ-LiAlO_2_ and Al_2_O_3_, respectively. The DQ–SQ spectrum of LLZO contains a diagonal signal at ∼10.5 ppm (in the SQ dimension), which is assigned to the LaAlO_3_ side-product signal (Figure 2d), where each Al is surrounded by six other Al sites at distances of approximately 3.8 Å^56^ (Table 1). A second signal is also seen at ∼79 ppm, but not at ∼68 ppm, indicating that the Al ions that give rise to the resonance at ∼79 ppm are in Al-rich local environments, but those that give rise to the ∼68 ppm resonance are not. This is again consistent with the assignment of the ∼79 ppm resonance to γ-LiAlO_2_.^55^

**Table 1:** Number of Nearest Al Neighbours for an Al Atom in an 24d Site along with the Distance (within ∼6 Å) between Them in γ-LiAlO_2_, LaAlO_3_, and Al-LLZO, Calculated from Their Respective ICSD Files and Shown in the Figure S4^a^

γ-LiAlO_2_		LaAlO_3_		Al-LLZO	
4 Al–Al	3.12 Å	6 Al–Al	3.79 Å	4 Al–Al 24d	3.98 Å
2 Al–Al	4.05 Å	6 Al–Al	5.35(62) Å	8 Al–Al 24d	6.07 Å
4 Al–Al	4.93 Å	6 Al–Al	5.36(46) Å		
4 Al–Al	5.17 Å				
4 Al–Al	5.34 Å				
4 Al–Al	5.42 Å				
2 Al–Al	5.68 Å				

a The number of Al neighbours and the distance between the Al neighbours along with their relaxation rates determine the intensity of the cross-peak in the DQ–SQ spectrum.

In order to rationalize why the ∼68 ppm signal was not seen in the LLZO DQ–SQ spectrum, the spatial proximity of Al dopants in this structure should be considered. Upon fitting the ^27^Al MAS NMR spectrum of Al-LLZO + 10% Li (Figure S2), it was found that about 45.4% of the total Al is present in the LaAlO_3_ impurity, and hence the rest of the Al should be incorporated into other phases. In the extreme case, if we assume that all of the residual Al is in the LLZO lattice, then about ∼1.57 Al atoms occupy each unit cell corresponding to a formula of Al_0.14_Li_6.58_La_3_Zr_2_O_12_ (i.e., there is a partially occupancy of 0.065 of Al in the 24d sites, as detailed in the Supporting Information). A 24d site has four nearby 24d sites, which are at a distance of 3.98 Å, Table 1. Thus, the probability that one Al in a 24d is nearby another Al in a 24d site is 0.065 × 4 (0.26). Since the DQ–SQ spectrum only couples two Al-nuclei that are both present in the |+1/2> or |−1/2> eigenstates, while the nearby Al nuclei have essentially an equal probability of being in one of the six eigenstates of the *I* = 5/2 nucleus, this further reduces the probability that one central transition ^27^Al spin is adjacent to another by 1/6. This means that the probability that one DQ–SQ observable Al-nucleus is next to another in a 24d site in LLZO is only 0.043. (In contrast, the probability rises to 0.66 for γ-LiAlO_2_). Thus, unless there is significant clustering of Al atoms, no, or extremely weak cross-peaks, are expected in the DQ–SQ spectra of LLZO.

DQ–SQ spectra were also recorded as a function of evolution/refocusing times (Figure S3), the intensity of the cross-peaks growing with evolution time, reaching a maximum at ∼4 rotor periods (95.2 μs) for LaAlO_3_, and ∼6 rotor periods (142.8 μs) for the resonance at ∼79 ppm assigned to γ-LiAlO_2_. The LaAlO_3_ cross-peak signal intensity dropped steadily after the maxima to zero, whereas the intensity of the cross-peak corresponding to γ-LiAlO_2_ decreased but was still present even after 16 rotor periods (380.8 μs). In addition, the maximum intensity of the cross-peak from the LaAlO_3_ was more intense than that from γ-LiAlO_2_, which is ascribed to the larger amount of LaAlO_3_ present in the sample. The more steady increase in the γ-LiAlO_2_ cross peak intensity vs that from LaAlO_3_ is ascribed to the difference in numbers and distances of the Al spins in the first cation coordination shells,—while an Al spin in γ-LiAlO_2_ has four nearby Al ions with a shorter Al–Al distance of 3.12 Å, it also has a further two at 4.05 Å. In contrast, the Al spins in LaAlO_3_ have six nearby Al nuclei in the first coordination cation shell at the longer distance of 3.79 Å (Table 1). The DQ/SQ relaxation rates of the two environments will also contribute to signal decay. The signal at ∼68 ppm is not seen even at longer mixing times; the second shell of 24d Al neighbors are more than 5.5 Å apart, and thus the dipolar coupling will be very weak between central and second shell Al nuclei.

Therefore, we assign the ∼79 ppm resonance to γ-LiAlO_2_, even though no evidence for this phase is seen in the SXRD pattern. The ∼68 ppm resonance is assigned to Al in the 24d site in the LLZO lattice in agreement with previous DFT studies, in which Al-substitution on this site was associated with the lowest energy.^36,41^

### 3.2 Effect of Li Excess in the Precursors on the ^27^Al MAS NMR Spectrum of Al-LLZO

**Figure 3:**
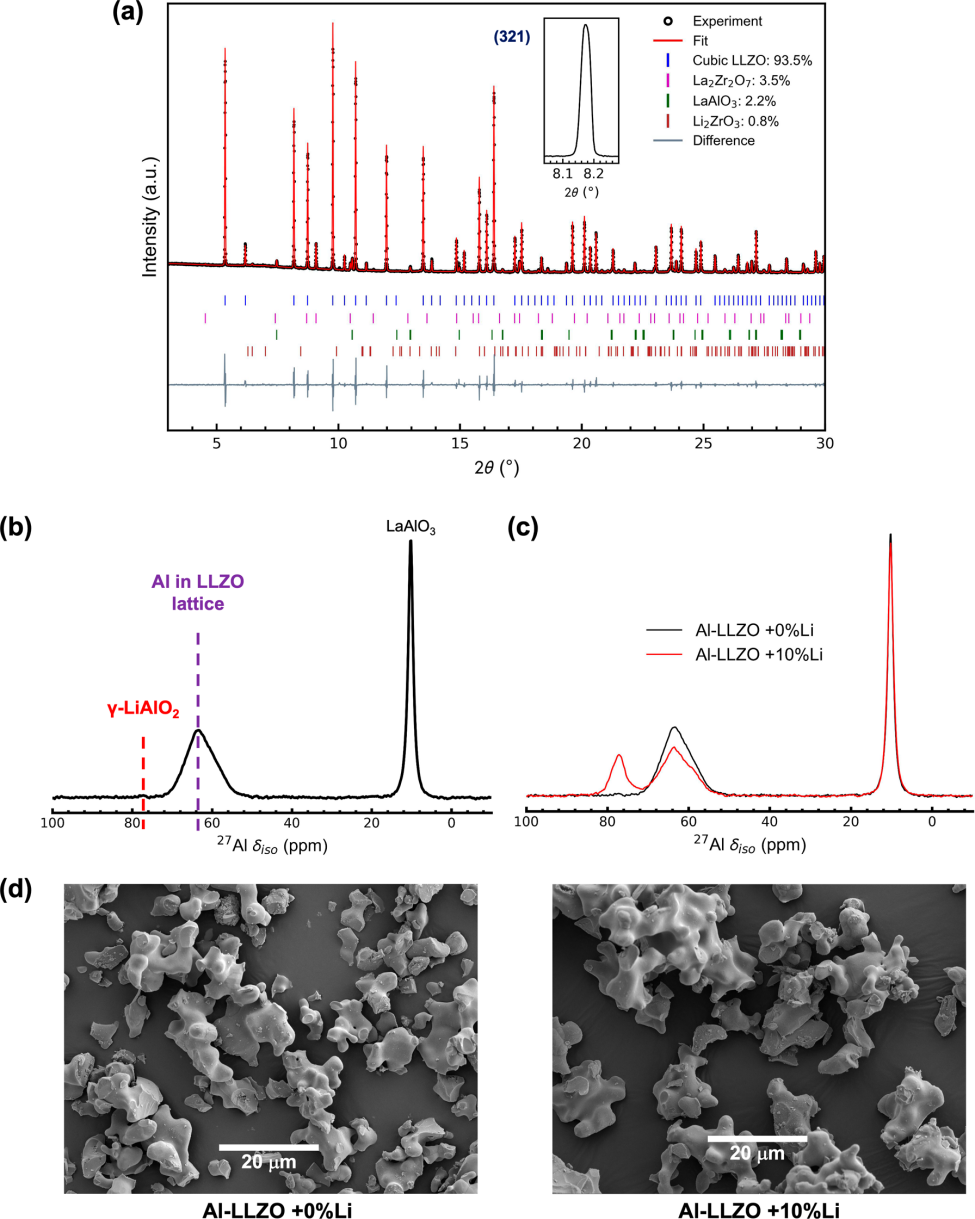
(a) SXRD pattern (λ = 0.493 Å) of Al-LLZO powder synthesized with no excess Li in the precursors (Al-LLZO + 0% Li) with an inset showing the (321) reflection of Al-LLZO. (b) ^27^Al MAS NMR spectrum of the Al-LLZO + 0% Li samples. (c) Comparison of ^27^Al MAS NMR spectra of the Al-LLZO + 10% Li and Al-LLZO + 0% Li powders. All spectra were normalized to respective sample weights during measurements. (d) Comparison of the SEM images of Al-LLZO + 10% Li and Al-LLZO + 0% Li powders.

To further test the hypothesis that γ-LiAlO_2_ forms during synthesis, Al-LLZO was synthesized with precursors with the exact stoichiometric of LLZO, i.e., with no excess Li (Al-LLZO + 0% Li), and its SXRD pattern is shown in Figure 3a.

**Figure 4:**
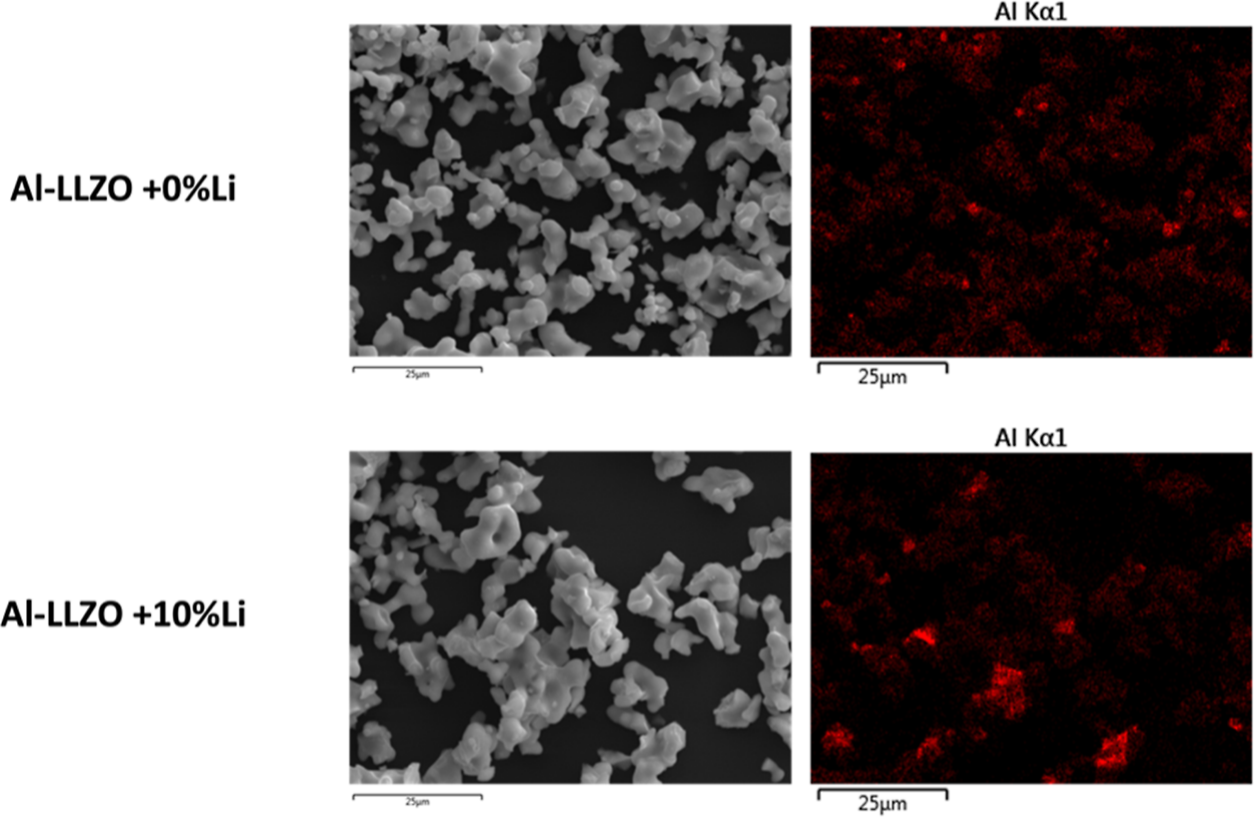
SEM–EDX map showing more heterogeneity in the Al in Al-LLZO + 10% Li sample as compared to the Al-LLZO + 0% Li sample.

A close inspection of the SXRD patterns of the Al-LLZO + 0% Li and Al-LLZO + 10% Li samples (Figures 2a and 3a) showed some tetragonal phase formation when excess Li is used, whereas no tetragonal phase was observed when excess Li was not used in the precursors. This is most likely due to sufficient incorporation of Al in the LLZO lattice for cubic phase formation when excess Li is not used, strongly suggesting some excess Li can be accompanied by the formation of lithium aluminates. Approximately 3.5% La_2_Zr_2_O_7_ pyrochlore was also observed in Al-LLZO + 0% Li (Figure 3a). In both the cases, small amounts of LaAlO_3_ and Li_2_Zr_2_O_3_ were observed, whereas γ-LiAlO_2_ was not observed even in these high-resolution SXRD patterns. SEM-EDS images of these samples shown in Figure 4 show significant heterogeneity in Al distribution in the Li-rich case (Al-LLZO + 10% Li) compared to the Li-poor case (Al-LLZO + 0% Li).

The ^27^Al MAS spectra of Al-LLZO + 10% Li and +0% Li are compared in Figure 3c. Both the ∼68 ppm (Al in LLZO) and ∼10.5 ppm (LaAlO_3_) resonances were observed in the ^27^Al MAS NMR spectrum of the two samples, but the ∼79 ppm resonance assigned to γ-LiAlO_2_ was not observed in Al-LLZO + 0% Li. Furthermore, the intensity of the resonance corresponding to Al in the LLZO lattice increased (Figure 3c), whereas the LaAlO_3_ resonance decreased slightly in intensity on adding excess Li. Given that the total amount of Al is the same in both samples, the slight decrease in LaAlO_3_ resonance intensity implies that more Al should have gone into other phases (either LLZO or γ-LiAlO_2_) in the Al-LLZO + 0% Li sample. If the ∼79 ppm resonance corresponded to an LLZO environment in which two or more Al were in close proximity (i.e., an environment that would be seen in the DQ–SQ 2D experiment), this resonance would likely have increased in intensity, rather than disappearing. Since no excess Li was used in the synthesis, all the Li was likely consumed during the formation of LLZO, and there was not enough Li available to drive γ-LiAlO_2_ formation.

The larger implication of these observations is that while some excess Li is necessary to synthesize cubic LLZO to compensate for Li loss at elevated temperatures (1000 °C in this study), any further excess Li will instead lead to unintentional tetragonal phase formation, heterogeneity in Al distribution, and γ-LiAlO_2_ in the final product. The formation energies of LiAlO_2_, Al-LLZO, and LLZO are very similar^57,58^ and are equally likely to form during synthesis. Thus, when excess lithium is used in the synthesis, LiAlO_2_ will form, and there will be a higher probability of tetragonal phase formation due to Al consumption.

The deleterious effect of excess Li in precursors resulting in tetragonal phase formation has recently been reported in the case of Ga-LLZO.^59^

### 3.3 Effect of Sintering on MAS NMR of Al-LLZO

**Figure 5:**
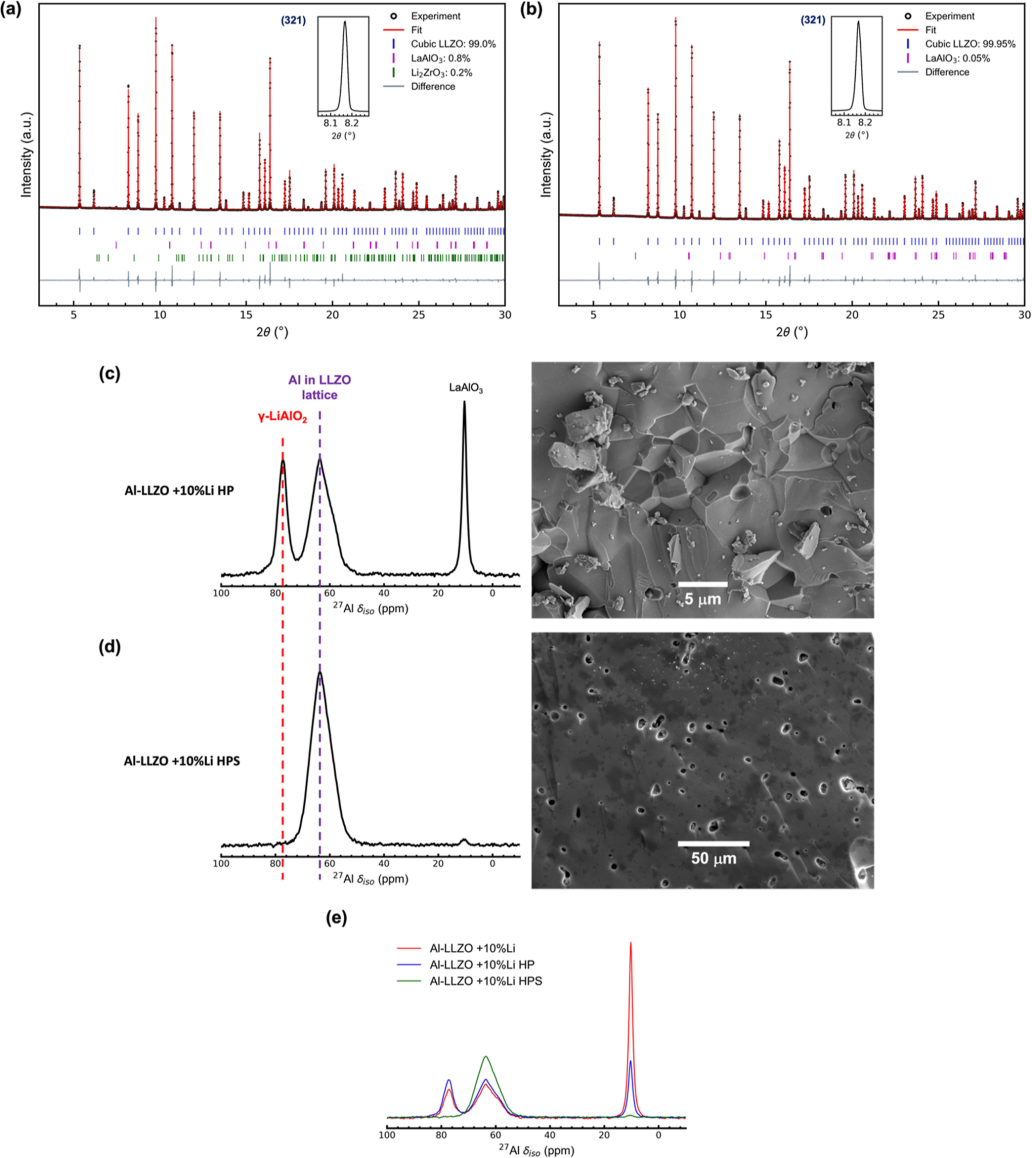
SXRD patterns (λ = 0.493 Å) with an inset showing the (321) LLZO reflection of (a) a hot-pressed sample of Al-LLZO + 10% Li: Al-LLZO + 10% Li HP and (b) hot-pressed Al-LLZO + 10% Li and further sintered for 12 h under O_2_ at 1200 °C: Al-LLZO + 10% Li HP further sintered. (c,d) shows the ^27^Al MAS NMR spectra and corresponding SEM images of Al-LLZO + 10% Li HP and Al-LLZO + 10% Li HPS respectively. (e) Comparison of ^27^Al MAS NMR spectra of the three different Al-LLZO powders.

To check whether γ-LiAlO_2_ still persists after sintering, the Al-LLZO powders (synthesized with 10% excess Li, Al-LLZO + 10% Li) were hot-pressed into pellets, which were then polished to remove any decomposition products (La_2_Zr_2_O_7_) on the surface and ground inside a glovebox. The SXRD pattern of the hot-pressed sample (Al-LLZO + 10% Li HP) is shown in Figure 5a. Rietveld refinement showed that the sample was composed mainly of cubic Al-LLZO with a reduced amount of side-products (LaAlO_3_ and Li_2_Zr_2_O_3_) as compared to the Al-LLZO + 10% Li sample. Moreover, the peaks corresponding to LLZO sharpened due to improved crystallinity, and the tetragonal phase disappeared, suggesting some redistribution of Al due to loss of Li during hot-pressing at elevated temperatures. Similar to the previous samples, the SXRD pattern did not show any sign of γ-LiAlO_2_. The SEM images of the cross-section of the pellet showed that Al-LLZO + 10% Li HP had a similar grain size as compared to the Al-LLZO + 10% Li sample (Figures 5c and 3d). A SEM-EDS map comparison showed that there was still some heterogeneity in the Al distribution, which may be due to the presence of LaAlO_3_ or γ-LiAlO_2_ (Figure S5).

The ^27^Al MAS NMR spectrum of the Al-LLZO + 10% Li HP sample is shown in Figure 5c. Three resonances corresponding to Al in γ-LiAlO_2_, the LLZO lattice, and LaAlO_3_ were observed, as in the Al-LLZO + 10% Li sample. Upon fitting the MAS NMR spectrum of Al-LLZO + 10% Li HP sample, it was found that only about ∼52.7% of Al used in precursors had gone into the lattice (Figure S8). On comparing the MAS NMR spectra of Al-LLZO + 10% Li HP with that of the +10% Li sample (Figure 5e), the γ-LiAlO_2_ and LLZO resonances increased in intensity, whereas that from LaAlO_3_ substantially reduced in intensity, consistent with the SXRD results. This suggests that Al from both γ-LiAlO_2_ and LaAlO_3_ enter the LLZO lattice during hot-pressing, helping to explain why no tetragonal phase was observed in the SXRD pattern of Al-LLZO + 10% Li HP.

Since γ-LiAlO_2_ was still present in the Al-LLZO + 10% Li HP sample, which had a similar grain size to the Al-LLZO + 10% Li powder, the Al-LLZO + 10% Li HP pellets were sintered to increase their grain size to check whether γ-LiAlO_2_ is still present. The Al-LLZO + 10% Li HP were sintered at 1200 °C for 18 h under flowing O_2_, and the resultant pellet (Al-LLZO + 10% Li HPS) was again polished to remove any decomposition products (La_2_Zr_2_O_7_) on the surface and was then ground inside the glovebox. Its SXRD pattern is shown in Figure 5b. Rietveld refinement showed that the sample was mainly composed of Al-LLZO and a very small amount of LaAlO_3_; again, no γ-LiAlO_2_ is seen. No grain boundaries were observed in the SEM image (Figure 5d) of the cross-section of the pellet, suggesting large grain sizes (>200 μm). The SEM-EDS map of a pellet cross-section showed uniform distribution of Al (Figure S5).

The ^27^Al MAS NMR spectrum of Al-LLZO + 10% Li HPS (Figure 5d) shows no evidence of the γ-LiAlO_2_ signal, and only a very small LaAlO_3_ signal was observed, consistent with the SXRD pattern. In addition, the intensity of the signal corresponding to Al in the LLZO lattice increased (Figure 5e) compared to Al-LLZO + 10% Li HP sample. Upon fitting the MAS NMR spectrum, it was found that almost all of the Al (∼99.1%) used in the precursors has gone into the lattice (Figure S8).

To confirm whether γ-LiAlO_2_ signal indeed disappears in any sample with large grain sizes, a sample was prepared using an alternate route by sintering nanosized Al-LLZO powder at 1200 °C for 12 h, which again resulted in large grains (∼200 μm) (see Supporting Information for more details and Figure S9). The γ-LiAlO_2_ signal was again not observed.

**Figure 6:**
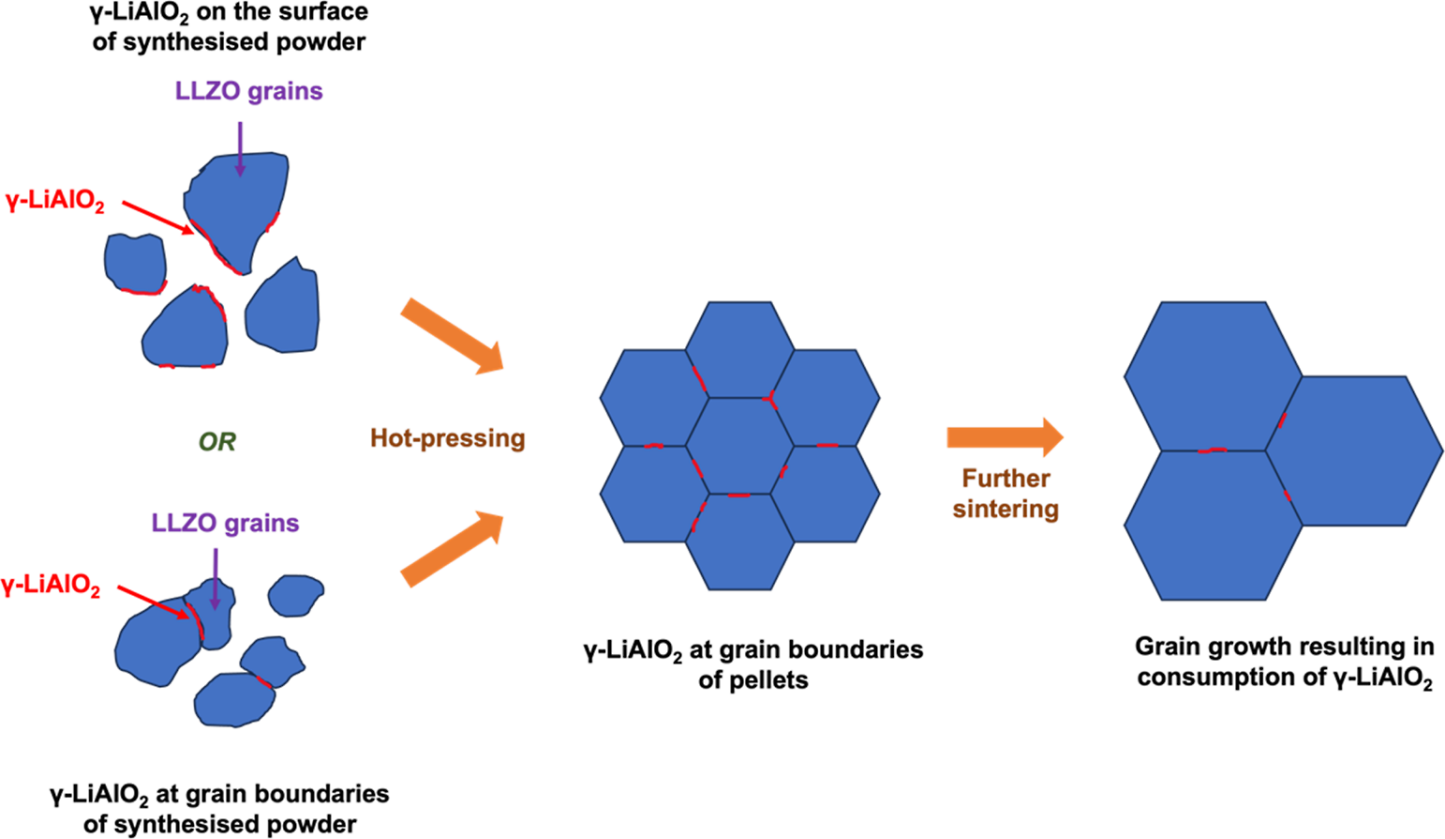
Representation of possible locations of γ-LiAlO_2_ (red) in LLZO grains (blue) and the possible reasons for its disappearance in samples with larger LLZO grain sizes.

The disappearance of the γ-LiAlO_2_ signal in samples with large grain sizes such as in Al-LLZO + 10% Li HPS sample and its presence in small-grained samples (Al-LLZO + 10% Li and Al-LLZO + 10% Li HP) can be explained if γ-LiAlO_2_ is present as a heterogeneous coating on the surface of grains or at the grain boundaries in the Al-LLZO + 10% Li sample. The absence of LiAlO_2_ in SXRD measurements suggests that this phase is either amorphous/poorly crystalline and/or is present at grain boundaries and hence has too short a coherence length to be observed even in the SXRD. As the sample grain size did not change drastically after hot-press sintering (Figures 3d and 5c), negligible changes are expected in the existing grain boundaries that contain γ-LiAlO_2_, and the new grain boundaries generated from the once free surface of grains in Al-LLZO + 10% Li powder will also contain γ-LiAlO_2_ (Figure 6). Thus, the ^27^Al MAS NMR spectrum is expected to be similar for Al-LLZO + 10% Li and Al-LLZO + 10% Li HP samples, as seen experimentally. When Al-LLZO 10% HP was further sintered to form pellets with large grain sizes (Al-LLZO + 10% Li HPS), any thin or amorphous layer of γ-LiAlO_2_ on the surface or in grain-boundaries of Al-LLZO + 10% Li HP would have been absorbed into the bulk of grains during sintering. The resulting samples have fewer grain boundaries and have too little γ-LiAlO_2_ to contribute to the ^27^Al MAS NMR spectrum, so the signal corresponding to γ-LiAlO_2_ is not seen in these samples (Figure 6).

The location of γ-LiAlO_2_, whether it is present in grain boundaries or at the surfaces of grains in Al-LLZO + 10% Li sample will need to be examined in greater detail in a future study.

### 3.4 Effect of γ-LiAlO_2_ on the Ionic Conductivity of the Sintered Samples

**Figure 7:**
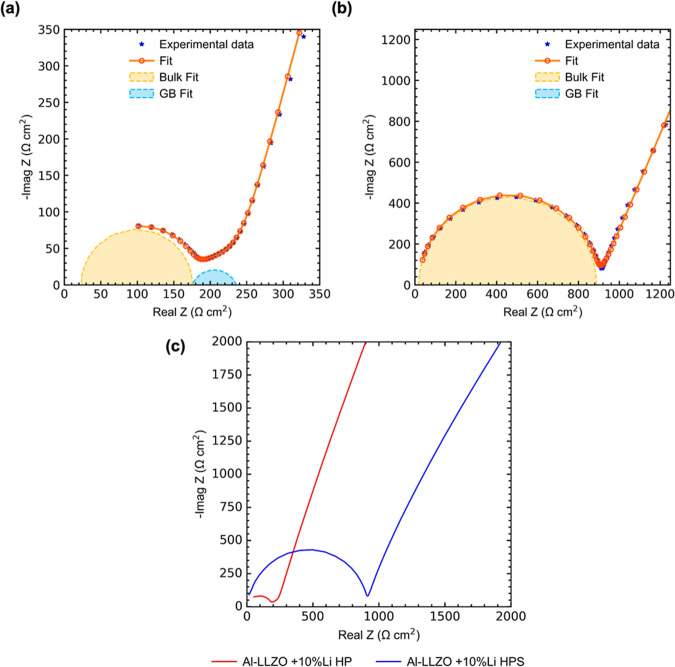
Blocking electrode impedance measurements of Al-LLZO at RT (a) hot-pressed Al-LLZO powder +10% Li powder, Al-LLZO 10% Li HP and (b) hot-pressed and further sintered powder from +10% Li powder, Al-LLZO + 10% Li HPS. The fit is shown along with the impedance plots. (c) Comparison of samples shown in (a,b).

To understand how the presence of γ-LiAlO_2_ affects the ionic conductivity of sintered samples, blocking electrode impedance measurements were done at RT on the two sintered samples, Al-LLZO + 10% Li HP, and Al-LLZO + 10% Li HPS (Figure 7a,b). The impedance plots show that the resistance of the Al-LLZO + 10% Li HPS sample is not simply the sum of bulk and grain boundary resistance of Al-LLZO + 10% Li HP sample (Figure 7c). Instead, the total resistance of the Al-LLZO + 10% Li HPS samples increases by about a factor of ∼6. The Al-LLZO + 10% Li HP sample showed two features corresponding to bulk and grain boundary resistance. On fitting, the samples showed a bulk conductivity of ∼0.66 mS cm^–1^ (and a total conductivity of ∼0.47 mS cm^–1^). By contrast the Al-LLZO + 10% Li HPS sample showed only one feature corresponding to the bulk resistance and upon fitting the spectra, a bulk conductivity of 0.11 mS cm^–1^ was obtained.

By using the intensities of the ^27^Al signals of the LLZO peaks in the MAS NMR spectra, the Al content in the LLZO samples can be calculated (as detailed in the Supporting Information) as approximately Al_0.2_Li_6.4_La_3_Zr_2_O_12_ for Al-LLZO + 10% Li HP sample and Al_0.36_Li_5.92_La_3_Zr_2_O_12_ for the Al-LLZO + 10% Li HPS sample. The increase in Al (and by extension decrease in Li) content in LLZO correlates with the decrease in ionic conductivity. It has been reported that ionic diffusion in LLZO occurs via multi-ion concerted migration mechanism, wherein approximately 3 ions move in correlated motion in the lattice.^60^ It was suggested that superionic conductivity can be activated by increasing mobile charge carrier concentration in solid electrolytes.

Thus, the drop in the conductivity of the Al-LLZO + 10% Li HPS sample is most likely due to the reduction in charge carrier concentration (Li) due to an increase in Al concentration as compared to the Al-LLZO + 10% Li HP sample. This is in line with a recent report wherein a reduction in bulk ionic conductivity of Al-LLZO was observed with increasing Al content in the precursors.^61^

The existence of Al in γ-LiAlO_2_ and the uneven distribution of Al between γ-LiAlO_2_ and the LLZO lattice depending on synthesis conditions and grain sizes might be one of the major reasons for the wide-ranging total ionic conductivity values reported in the literature. The absence of tetragonal Al-LLZO in the Al-LLZO + 10% Li HP sample suggests that ∼0.19 Al per 7 Li atoms (quantified from MAS NMR spectra see Supporting Information) is enough Al to form cubic LLZO, and the highest conductivity is found at the transition point between tetragonal and cubic LLZO.

### 3.5 ^71^Ga MAS NMR and EIS Analysis of Ga-LLZO

**Figure 8:**
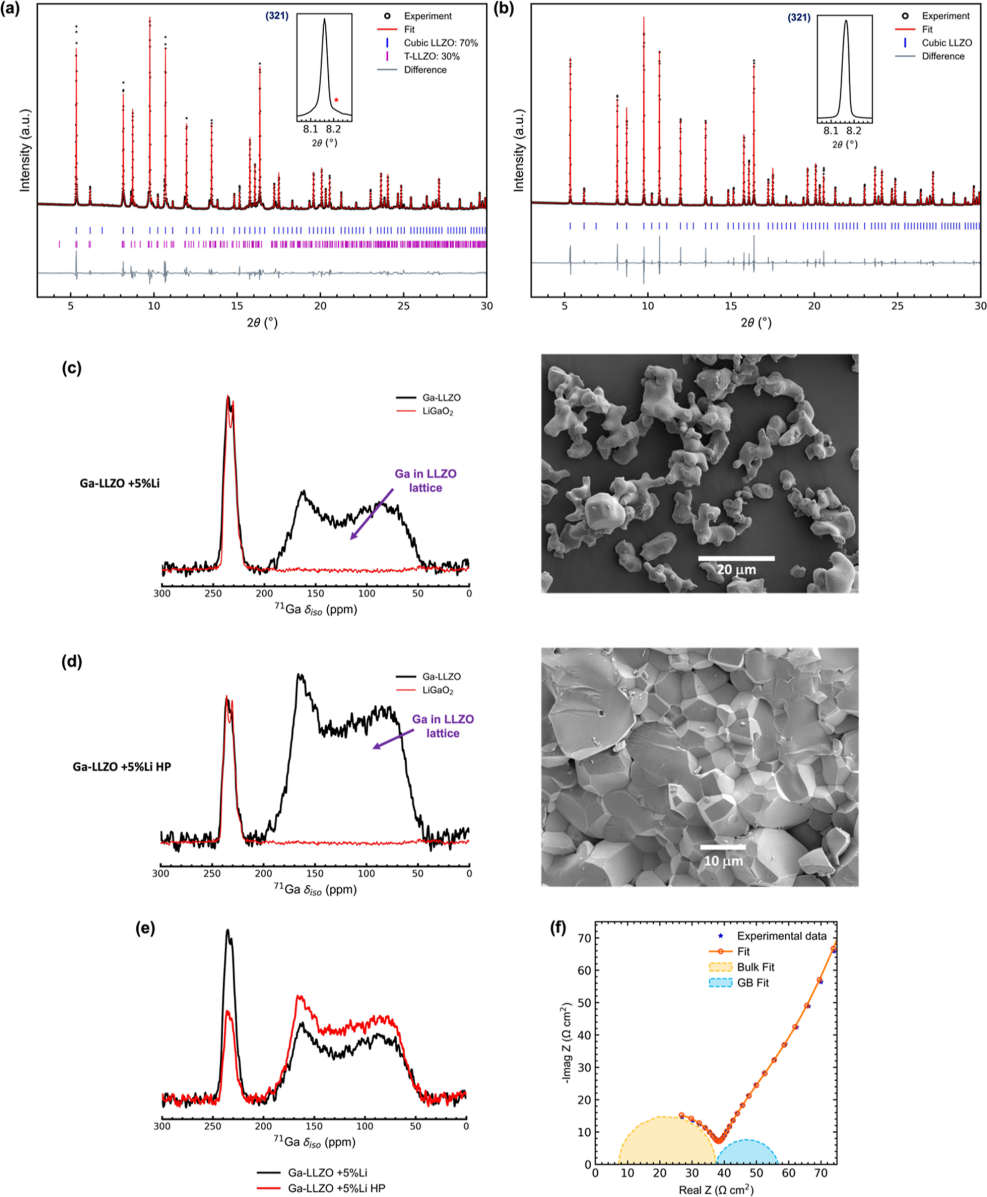
(a) SXRD patterns (λ = 0.493 Å) of (a) Ga-LLZO + 5% Li and (b) hot-pressed sample of Ga-LLZO + 5% Li (Ga-LLZO + 5% Li HP) with an inset showing the peak corresponding to (321) reflection where *indicates tetragonal LLZO phase. The ^71^Ga MAS NMR spectra of (c) Ga-LLZO + 5% Li and (d) Ga-LLZO + 5% Li HP (overlaid with the ^71^Ga MAS NMR spectrum of LiGaO_2_) and their corresponding SEM images. (e) Comparison of ^71^Ga MAS NMR spectra of both samples. Both spectra were normalized to respective sample weights. (f) Blocking electrode impedance measurement of the Ga-LLZO + 5% Li HP sample.

**Figure 9:**
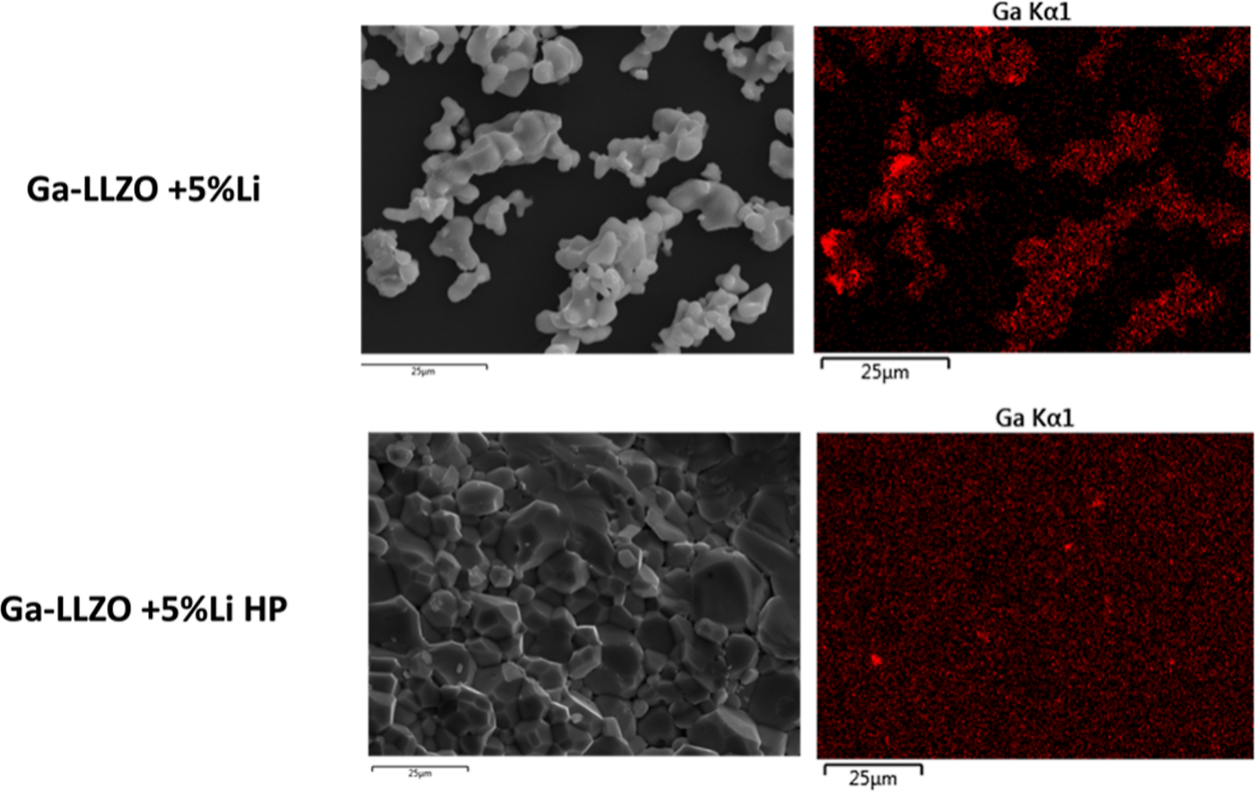
SEM–EDX map of Ga-LLZO + 5% Li and Ga-LLZO + 5% Li HP showing regions of Ga enrichment in both samples.

Similar issues exist concerning the lattice site occupied by Ga dopant in Ga-LLZO. Ga-LLZO (Ga_0.2_Li_6.3_La_3_Zr_2_O_12_) was synthesized with 5% Li excess (Ga-LLZO + 5% Li) in the precursors and its SXRD pattern is shown in Figure 8a. Since excess Li in the precursors resulted in γ-LiAlO_2_ side-product in Al-LLZO, only 5% excess Li was used in the synthesis of Ga-LLZO. Rietveld refinement showed that the sample contained ∼30% tetragonal phase alongside the cubic phase and no other side-product could be identified. The SEM-EDS mapping of the synthesized powder showed regions which were rich in Ga and deficient in La and Zr (Figures 9 and S13).

^71^Ga MAS NMR spectrum of the synthesized sample showed two distinct resonances (Figure 8c) as has been previously observed in the literature.^37,41,45^ Since, ^71^Ga is an (*I* = 3/2) quadrupolar nucleus, the spectrum was fitted and the isotropic chemical shifts, ∼ 199 ppm and ∼242 ppm were extracted. ^71^Ga in tetrahedral environments (GaO_4_) has been reported to have shifts around 150–250 ppm.^62^ Therefore, the two resonances observed can be attributed to Ga in two distinct tetrahedral environments. LiGaO_2_ was synthesized (SXRD pattern in Figure S10) and its ^71^Ga MAS NMR spectrum was collected and again overlaid with that of Ga-LLZO (Figure 8c). As in the case of Al-LLZO, the match between LiGaO_2_ and the ∼242 ppm resonance observed in Ga-LLZO was very good with similar quadrupolar coupling constants (3.84 ± 0.10 and 3.82 ± 0.10 MHz for LiGaO_2_ and Ga-LLZO respectively) and asymmetry parameters (0.41 ± 0.02 and 0.40 ± 0.02 for LiGaO_2_ and Ga-LLZO respectively) being found for the two materials. The existence of LiGaO_2_ in the LLZO samples can explain the La and Zr deficient and Ga rich regions seen by SEM-EDS. The remaining ∼199 ppm resonance can be attributed to Ga in the 24d site in the LLZO lattice.

To check whether LiGaO_2_ persisted after sintering, the synthesized Ga-LLZO + 5% Li powder was hot pressed (Ga-LLZO + 5% Li HP) and its SXRD pattern is shown in Figure 8b. Rietveld refinement showed that the sample was composed entirely of cubic phase LLZO. The SEM images of the cross-section of the Ga-LLZO + 5% Li HP pellet showed that it had grains of similar size as in the Ga-LLZO + 5% Li sample (Figure 8c,d) and the EDS map showed similar La and Zr deficient and Ga rich regions as in the Ga-LLZO + 5% Li sample (Figures 9 and S13). The ^71^Ga MAS NMR spectrum of the Ga-LLZO + 5% Li HP sample showed two resonances with identical isotropic chemical shifts as those in the Ga-LLZO + 5% Li sample i.e., ∼199 ppm (Ga in LLZO lattice) and ∼242 ppm (LiGaO_2_). These observations are consistent with the recent observation of LiGaO_2_ in sintered Ga-LLZO pellets by high resolution transmission electron microscopy.^63^ The present work suggests that LiGaO_2_ is present not just in sintered pellets but also in the synthesized powders of Ga-LLZO.

A comparison of the two ^71^Ga MAS NMR spectra (Figure 8e) showed that upon hot-pressing, the intensity of the ∼242 ppm (LiGaO_2_) resonance was reduced while the intensity of the ∼199 ppm resonance (Ga in LLZO lattice) increased indicating that some Ga incorporates into the LLZO lattice from LiGaO_2_ leading to complete cubic phase formation (as seen by SXRD).

The ionic conductivity of the hot-pressed pellets was measured by EIS. In the blocking electrode impedance plot (Figure 8f), the hot-pressed samples showed two features corresponding to bulk and grain boundary resistance. On fitting, a bulk ionic conductivity of 2.8 mS cm^–1^ and a total ionic conductivity of 1.75 mS cm^–1^ was obtained which is among the highest reported total and bulk ionic conductivities for Ga-LLZO.^20,44^ A similar dependence of Li-ion conductivity on the Ga content in the LLZO lattice can be expected as in the case of Al-LLZO.

### 3.6 ^27^Al and ^71^Ga MAS NMR of LiAl_0.5_Ga_0.5_O_2_

**Figure 10:**
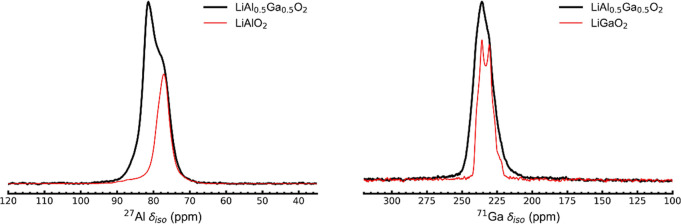
^27^Al (left) and ^71^Ga (right) MAS NMR spectra comparison of γ-LiAlO_2_ and LiGaO_2_ with LiAl_0.5_Ga_0.5_O_2_.

It has been reported in the literature that upon simultaneous doping of LLZO with Al and Ga, the resonance with higher chemical shift moves to higher frequencies in the ^27^Al MAS NMR spectrum while the resonance with higher chemical shift broadens in ^71^Ga MAS NMR spectrum.^37,41^ Since codoping in LLZO may also result in substitution of Al into LiGaO_2_ and vice versa, LiAl_0.5_Ga_0.5_O_2_ was prepared by mixing the precursors needed for LiAlO_2_ and LiGaO_2_ in a 1:1 ratio and heating at conditions similar to those used to prepare LLZO. The SXRD pattern of the resultant sample is two-phase comprising of both γ-LiAlO_2_ and LiGaO_2_, but with shifted peak positions suggesting that some Al was incorporated into LiGaO_2_ and vice versa (Figure S14). The ^27^Al MAS NMR spectrum showed shifting and broadening of the resonance toward higher frequencies compared to the undoped case (Figure 10) whereas the ^71^Ga MAS NMR spectrum showed broadening of the resonance (along with a small shift). Co-doped LLZO (Al_0.18_Ga_0.18_Li_5.92_La_3_Zr_2_O_12_) was synthesized and its corresponding ^27^Al and ^71^Ga MAS NMR were recorded and compared with LiAlO_2_/LiGaO_2_ and LiAl_0.5_Ga_0.5_O_2_. As expected, the higher resonance matched perfectly with LiAl_0.5_Ga_0.5_O_2_ (Figure S15). This further confirms that the resonance with higher chemical shift in both ^27^Al and ^71^Ga MAS NMR spectra of LLZO codoped with Al and Ga is due to the side products, γ-LiAlO_2_ or LiGaO_2_ with Ga and Al incorporation, respectively.

A recent NMR and DFT study by some of us suggested that the Li configuration in the first cation coordination shell around the dopant influences the dopants’ isotropic chemical shift,^41^ the more symmetric Al/Ga environment surrounded by 4 Li ions being associated with higher shifts and much smaller quadrupolar coupling constants. On this basis, the ∼79 and ∼242 ppm resonances in the ^27^Al and ^71^Ga MAS NMR spectra respectively were assigned to these configurations. The ∼79 ppm resonance was not, however, seen in the sample prepared in this work with no excess Li (Al-LLZO + 0% Li), which has a higher Al dopant level (and hence less Li; composition Al_0.36_Li_5.92_), but it was seen in the sample prepared with 10% excess Li (Al-LLZO + 10% Li) which has less Al (and more Li; composition Al_0.2_Li_6.4_ assuming the high frequency resonance originates from Al in LLZO).

The samples with higher Li contents are more likely to contain Al configurations with 4 Li around them and thus the resonance from this configuration should indeed have been more pronounced in the Al-LLZO + 10% Li sample. However, the prior study also saw the higher frequency resonance in other higher content Al samples, and no obvious trend between Al/Ga content and the intensity of this resonance was observed. Of note, this study did not directly account for the effect of Li mobility on the spectra and at RT, due to the Li motion around the dopants,^31,64^ the effect of different Li configurations on the dopants’ spectral properties is not directly observed. However, at very low temperatures, where the Li motion is hindered, ordering of Li around the dopant might influence the isotropic chemical shifts of the dopants, and additional broadening of the LLZO resonances might be expected. At room temperature, a weighted time-average of the spectra from the different configurations should result.

## 4 Conclusions

In this work, we have shown that Al and Ga dopants occupy only the 24d site in LLZO lattice putting rest to the speculation regarding dopant site occupancy in the battery community. The additional higher frequency resonance observed in this work and in previous studies in both ^27^Al and ^71^Ga MAS NMR spectra of Al- and Ga-LLZO has been identified as being due to the side-products, γ-LiAlO_2_/LiGaO_2_. These side products have been found to exist even in hot-pressed Al-LLZO and Ga-LLZO samples. Since γ-LiAlO_2_ and LiGaO_2_ are not observed in the SXRD patterns of LLZO samples, their presence has likely been overlooked by the community (including us).

The distribution of dopants between these side-products and the LLZO lattice has been found to considerably affect the ionic conductivity of LLZO. A decrease in ionic conductivity with increase in dopant concentration in LLZO lattice was observed, which is ascribed to the reduction in charge carrier concentration as the amount of dopant increases. Thus, to achieve high ionic conductivity in LLZO, it is necessary to identify the minimum dopant concentration (and hence maximum Li content) required for the tetragonal to cubic phase transition through careful synthesis of LLZO. Finally, it is emphasized that some excess Li is needed to synthesize pure cubic phase LLZO at high temperatures (1000 °C) to account for Li loss, but any further excess Li will lead to the formation Al or Ga-containing side-products, resulting in the formation of the poorly ionically conducting tetragonal phase LLZO formation. The results from this study further suggest that the Li-excess content in the precursors can affect the dopant content in solid-state electrolytes (and thus its structure) due to the formation of lithium and dopant containing side-products. Li excess content in the precursors can also influence the extent of Li/transition metal mixing in other Li containing compounds such as LiNiO_2_, Ni-rich and NMC cathodes, thus the amount of Li excess during synthesis needs to be carefully chosen and its effect on synthesised products needs to be carefully studied.

## Supporting Information Available

SXRD of samples, fitting data of MAS NMR spectra, additional DQSQ plots, SEM-EDS maps, experimental methods (PDF)
