# Supplementary material for: Clarifying the Dopant Local Structure and Effect on Ionic Conductivity in Garnet Solid-State Electrolytes for Lithium-Ion Batteries
Source: Chem Mater. 2023 Nov 14;35(22):9632–46. doi: 10.1021/acs.chemmater.3c01831 (PMC10687891; doi:10.1021/acs.chemmater.3c01831)
Supplement: Supplementary file 1 — cm3c01831_si_001.pdf [file cm3c01831_si_001.pdf]

Clarifying the dopant local structure and effect on ionic conductivity in garnet solid-state electrolytes for lithium-ion batteries

Sundeep Vema[1,2], Astrid H. Berge[1], Supreeth Nagendran[1], Clare P. Grey[1,2,*]

[1] Yusuf Hamied Department of Chemistry, University of Cambridge, Lensfield Road, Cambridge, CB2 1EW, United Kingdom

[2] The Faraday Institution, Quad One, Harwell Campus, Didcot, OX11 0RA, United Kingdom

Supporting Information

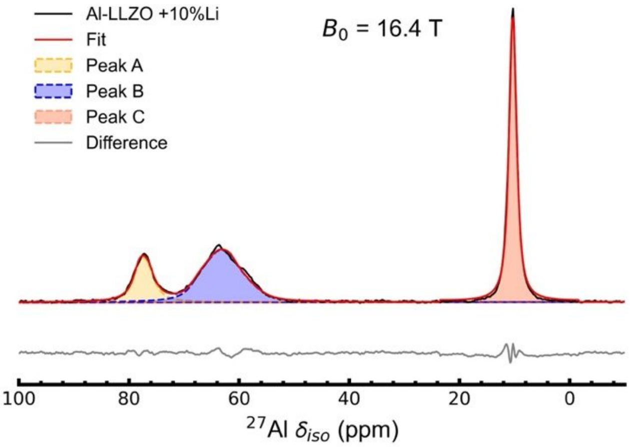

Fitted component	δ_{iso} [ppm]	C_Q [MHz]	η_Q	Intensity %
Peak A	79 ± 1	3.1 ± 0.5	0.1 ± 0.1	16.6
Peak B	68 ± 1	5.3 ± 0.5	0.6 ± 0.1	38.0
Peak C	11 ± 1	1.9 ± 0.5	0.1 ± 0.1	45.4

Figure S1: ^{27}Al MAS NMR spectrum (black curve) of Al-LLZO powder sample prepared with 10% excess Li in precursors, Al-LLZO +10%Li. The golden curve indicates the peak A, the blue curve indicates the peak B, and the orange curve indicates the peak C. The red curve indicates the overall fit and the grey curve indicates the difference between the fitted and observed data. The table shows the fitted ^{27}Al MAS NMR parameters (isotropic chemical shifts δ_{iso}, quadrupolar coupling constant C_Q and quadrupolar asymmetry parameter η_Q).

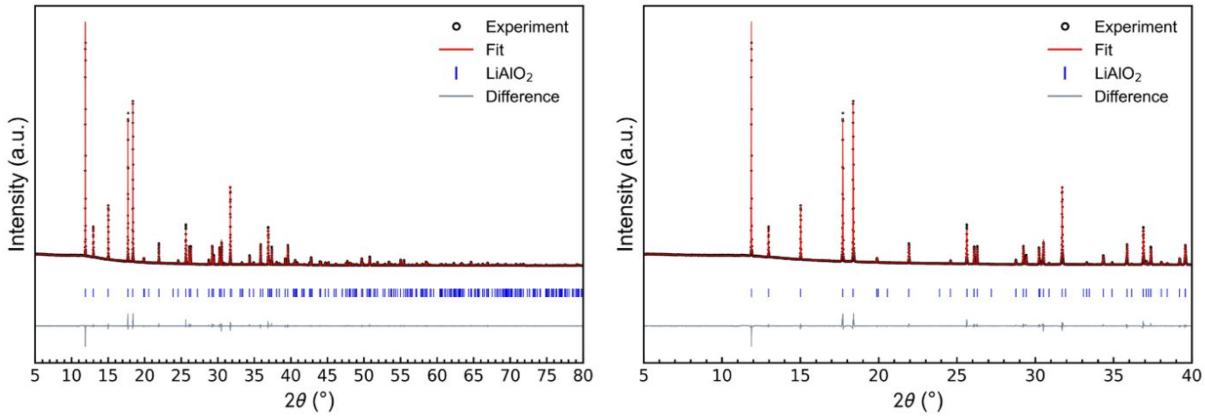

Figure S2: SXRD pattern (black circles) and calculated patterns from Rietveld refinement (red curve) of the synthesised γ-$LiAlO_2$ sample (λ = 0.824978 Å). The dark blue tick marks indicate the reflections from the $P4_12_12$ γ-$LiAlO_2$ phase. The grey curve indicates the difference between the fitted and the observed data. For this sample the refined lattice parameters were a and b = 5.168 Å and c = 6.269 Å. No side-products were detected in the XRD pattern.

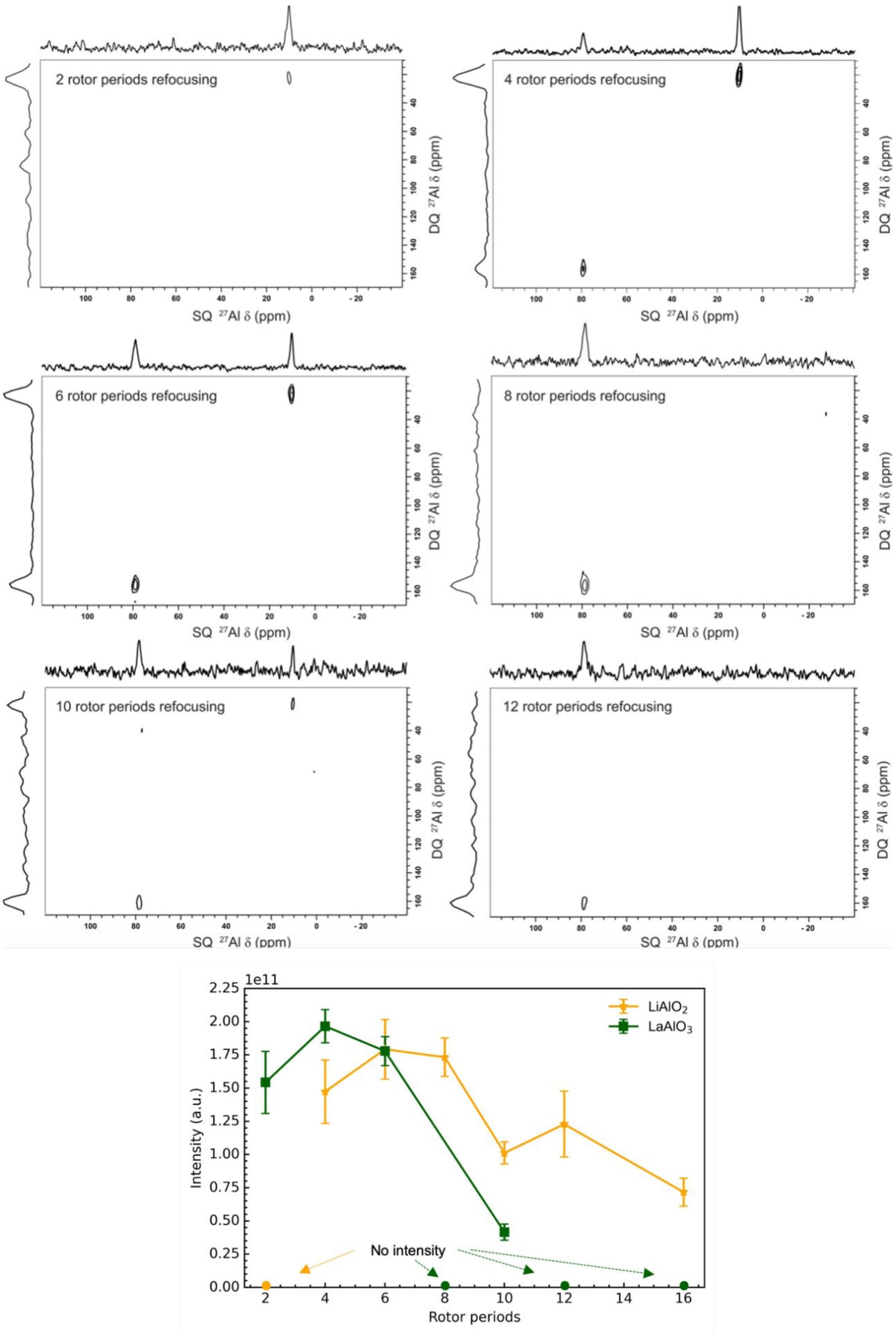

Figure S3: DQ-SQ 2D spectra (23.5T, 42kHz) for the same Al-LLZO +10%Li excess sample recorded with various refocusing times. A BR2_2^1 recoupling sequence was used to probe the dipolar through space interactions between the ^{27}Al nuclei. The intensity of each of the cross-peaks is shown as a function of the number of rotor period used for recoupling.

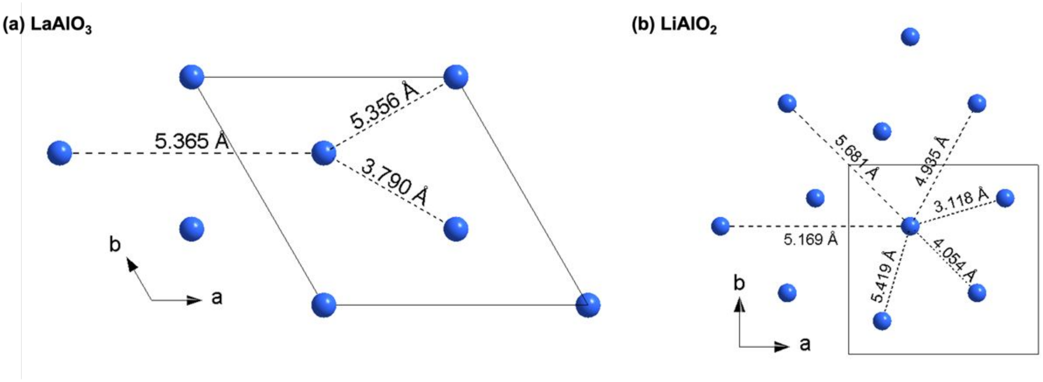

Figure S4: Representation of first local coordination shell in $LaAlO_3$ and $LiAlO_2$ along with distances between two Al atoms.

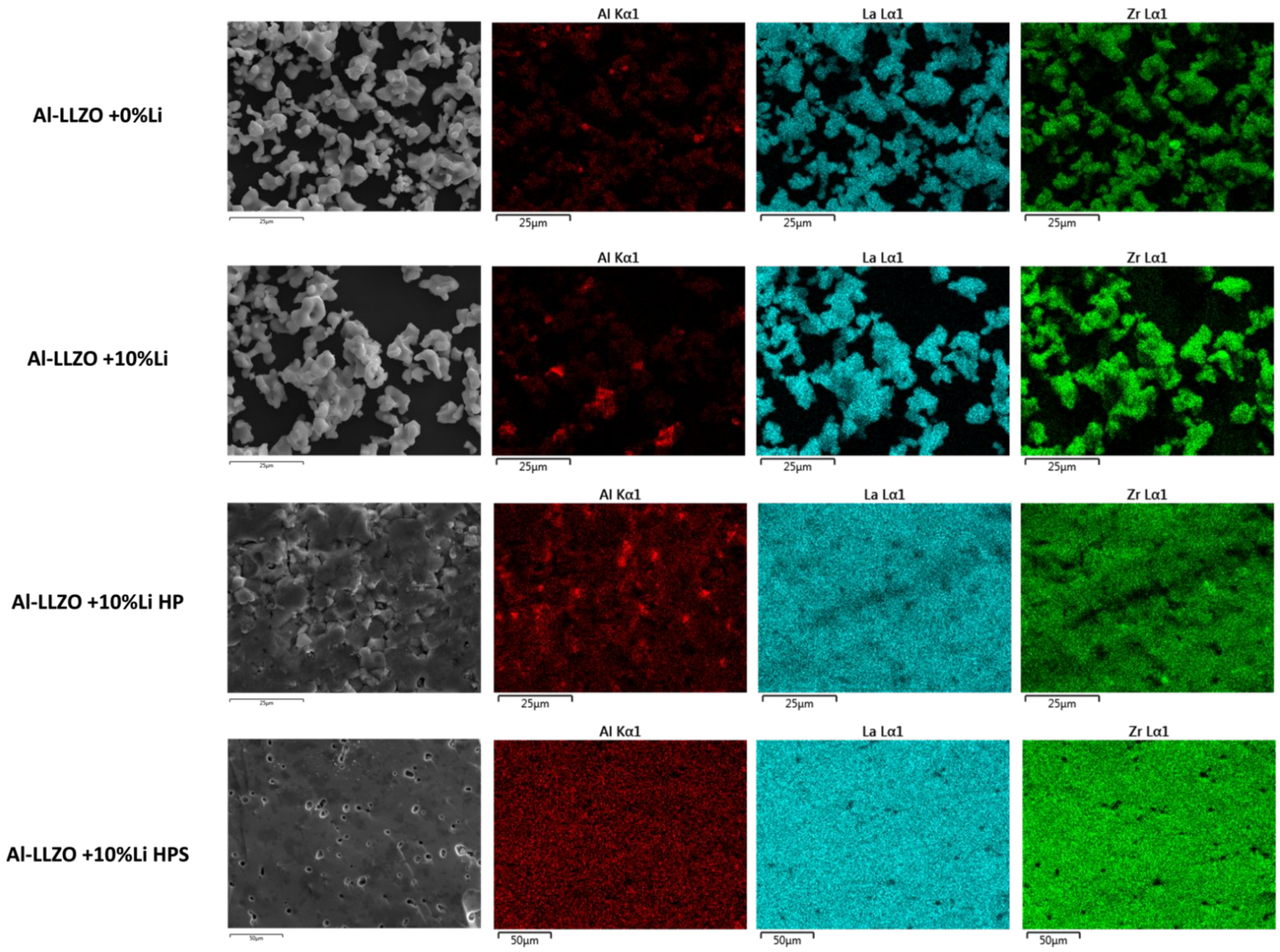

Figure S5: SEM-EDS maps Al-LLZO powders prepared under different conditions.

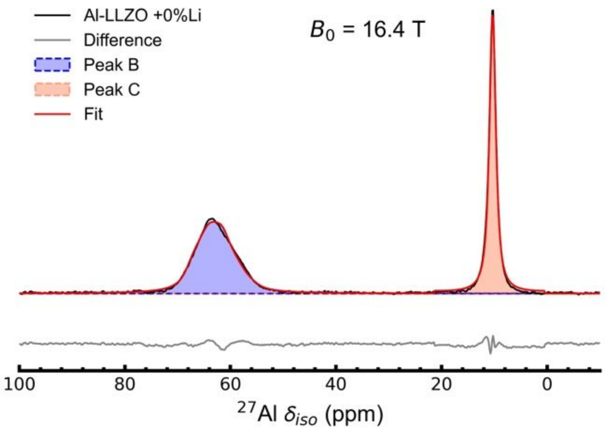

Fitted component	δ_{iso} [ppm]	C_Q [MHz]	η_Q	Intensity %
Peak B	68 ± 1	5.3 ± 0.5	0.6 ± 0.1	55.1
Peak C	11 ± 1	1.5 ± 0.5	0.1 ± 0.1	44.9

Figure S6: ^{27}Al MAS NMR spectrum (black curve) of Al-LLZO powder sample prepared with 0% excess Li in precursors, Al-LLZO +0%Li. The blue curve indicates the peak B, and the orange curve indicates the peak C. The red curve indicates the overall fit and the grey curve indicates the difference between the fitted and observed data. The table shows the fitted ^{27}Al MAS NMR parameters (isotropic chemical shifts δ_{iso}, quadrupolar coupling constant C_Q and quadrupolar asymmetry parameter η_Q).

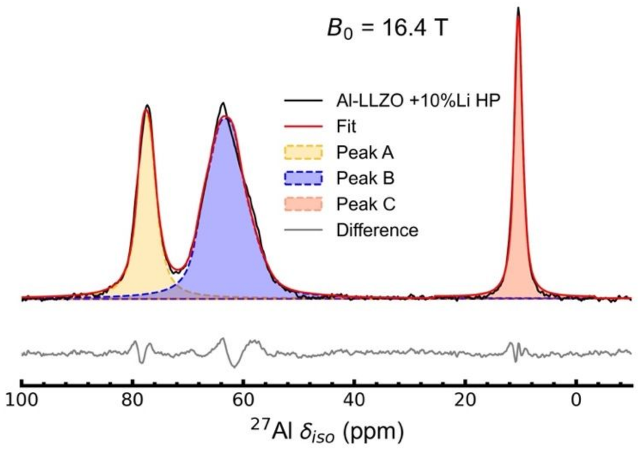

Fitted component	δ_{iso} [ppm]	C_Q [MHz]	η_Q	Intensity %
Peak A	79 ± 1	3.2 ± 0.1	0.1 ± 0.1	27.2
Peak B	68 ± 1	5.3 ± 0.5	0.6 ± 0.1	52.7
Peak C	11 ± 1	2 ± 0.5	0.1 ± 0.1	20.1

Figure S7: ^{27}Al MAS NMR spectrum (black curve) of hot-pressed Al-LLZO +10%Li sample, Al-LLZO +10%Li HP. The golden curve indicates the peak A, the blue curve indicates the peak B, and the orange curve indicates the peak C. The red curve indicates the overall fit and the grey curve indicates the difference between the fitted and observed data. The table shows the fitted ^{27}Al MAS NMR parameters (isotropic chemical shifts δ_{iso}, quadrupolar coupling constant C_Q and quadrupolar asymmetry parameter η_Q).

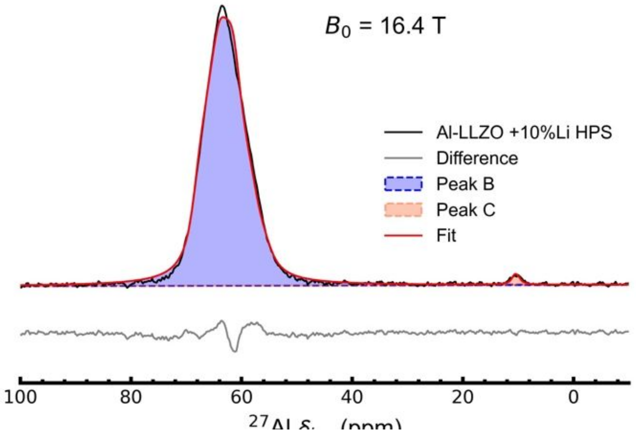

Fitted component	δ_{iso} [ppm]	C_Q [MHz]	η_Q	Intensity %
Peak B	68 ± 1	5.4 ± 0.5	0.6 ± 0.1	99.1
Peak C	11 ± 1	2 ± 0.5	0.1 ± 0.1	0.9

Figure S8: ^{27}Al MAS NMR spectrum (black curve) of further sintered Al-LLZO +10%Li HP sample, Al-LLZO +10%HPS. The blue curve indicates the peak B, and the orange curve indicates the peak C. The red curve indicates the overall fit and the grey curve indicates the difference between the fitted and observed data. The table shows the fitted ^{27}Al MAS NMR parameters (isotropic chemical shifts δ_{iso}, quadrupolar coupling constant C_Q and quadrupolar asymmetry parameter η_Q).

Calculation of Al content in Al-LLZO:

The precursors contain enough Al to obtain the composition $Al_{0.36}Li_{5.92}La_3Zr_2O_{12}$. If the fit to ^{27}Al MAS NMR spectrum of an Al-LLZO sample shows that x% of Al goes into the LLZO lattice, then the effective formula of LLZO becomes: $Al_{0.36x/100}Li_{7-1.08x/100}La_3Zr_2O_{12}$. Using this formula, the Al-LLZO samples used in this study had the following formula units: Al-LLZO 10%: ~$Al_{0.14}Li_{6.58}La_3Zr_2O_{12}$; Al-LLZO 0%: ~$Al_{0.2}Li_{6.4}La_3Zr_2O_{12}$; Al-LLZO 10% hot-pressed: $Al_{0.19}Li_{6.43}La_3Zr_2O_{12}$ and further sintered Al-LLZO 10% hot-pressed: $Al_{0.36}Li_{5.92}La_3Zr_2O_{12}$

Synthesis of large grained sample by conventional sintering method:

Pellets with large grain sizes were prepared by a conventional high temperature sintering method. The synthesised doped LLZO powders were wet milled in 10 gm batches with cyclohexane in a Restch PM100 Ball Mill (~60 mL of 2 mm Ø zirconia balls, Toray Ceram, Japan, in 125 mL Retsch Zirconia jar) at 400 rpm for 20 hours (10 min active mixing and 20 min rest cycle). The nano-sized powder was separated from Zirconia balls by dry milling for 5 mins at 400 rpm followed by sonication in Cyclohexane, sieving and drying the slurry over a hotplate. The process of dry milling for 5 mins and sieving was repeated 3-4 times. The sieved nano-sized powders were stored in airtight vials in a desiccator. The SEM image of the nano-sized powders is shown in Figure S7. The nano-sized powders were then isostatically pressed (EPSI cold isostatic press) in custom made latex rubber moulds, 7 mm Ø (Inner diameter), at 200 MPa for 2 mins. The pressed pellets were transferred to MgO crucibles (8 mm Ø I.D.) with MgO caps and covered with non-wet-milled mother powder and heated in a box furnace at 1200 °C for 12 hours with a 10 °C/min heating rate and a 10 °C/min cooling rate and transferred into a glovebox above 150 °C. The samples were dry polished and stored in a glovebox.

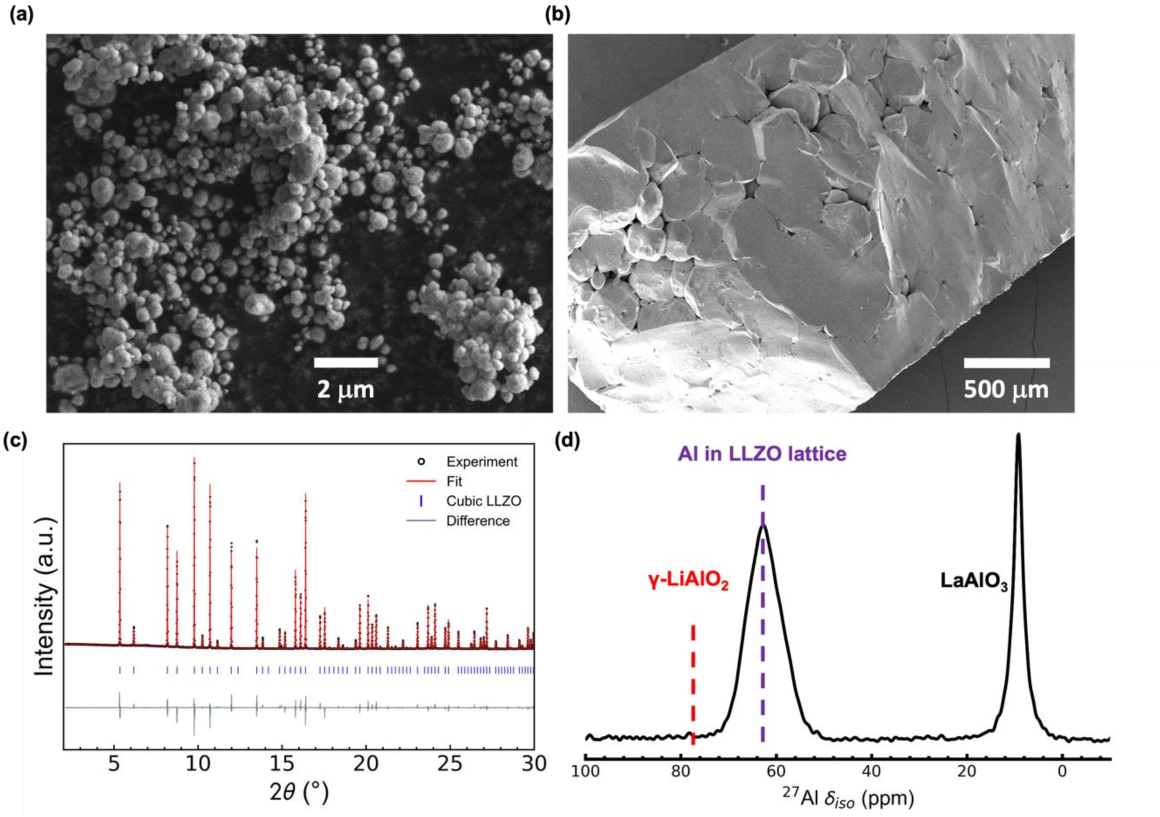

Figure S9: (a) SEM image of cyclohexane wet-ball milled Al-LLZO powder (b) SEM image of cross-section of the sintered Al-LLZO pellet (c) XRD pattern of the sintered Al-LLZO pellet (0.49381 Å) (d) ^{27}Al MAS NMR spectrum of ground powder of polished pellet inside glovebox

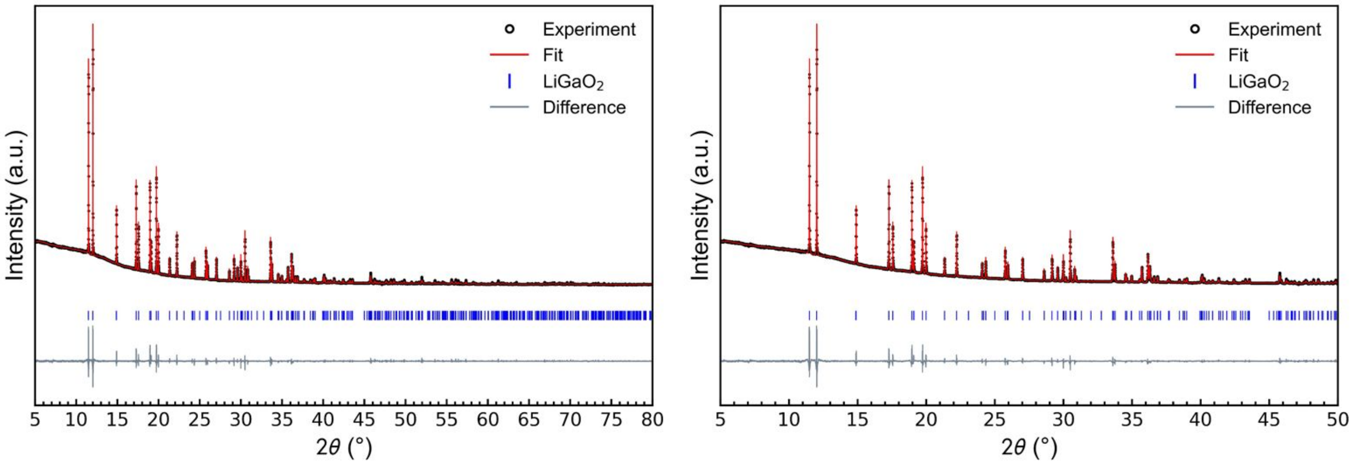

Figure S10: Raw XRD data (black circles) and calculated patterns from Rietveld refinement (red curve) of the $LiGaO_2$ sample (λ = 0.824978 Å). The dark blue tick marks indicate the reflections from $Pna2_1$ $LiGaO_2$ phase. The grey curve indicates the difference between the fitted and the observed data. For this sample the refined lattice parameters were a = 5.405 Å, b = 6.378 Å and c = 5.012 Å. No side-products were detected in the XRD pattern.

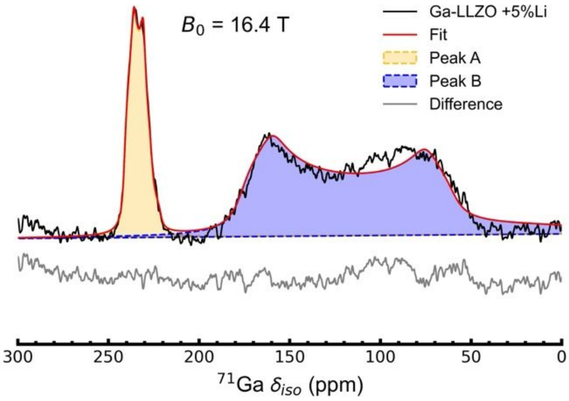

Fitted component	δ_{iso} [ppm]	C_Q [MHz]	η_Q	Intensity %
Peak A	242 ± 1	3.8 ± 0.1	0.4 ± 0.1	24.4
Peak B	199 ± 1	12 ± 0.5	0.1 ± 0.1	75.6

Figure S11: ^{71}Ga MAS NMR spectrum of Ga-LLZO powder sample prepared with 5% excess Li in precursors at 16.4 T with a MAS speed of 40 kHz (black curve) along with fitting. The golden curve indicates the peak A, and blue curve indicates the peak B. The red curve indicates the overall fit and the grey curve indicates the difference between the fitted and observed data. The table shows the fitted ^{71}Ga MAS NMR parameters (isotropic chemical shifts δ_{iso}, quadrupolar coupling constant C_Q and quadrupolar asymmetry parameter η_Q).

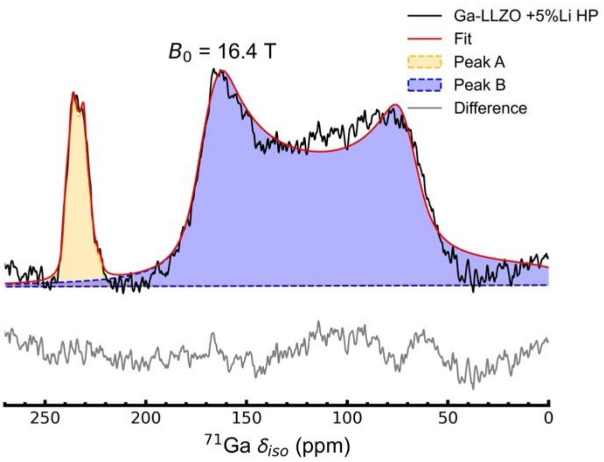

Fitted component	δ_{iso} [ppm]	C_Q [MHz]	η_Q	Intensity %
Peak A	242 ± 1	3.8 ± 0.1	0.4 ± 0.1	10.7
Peak B	199 ± 1	12 ± 0.5	0.1 ± 0.1	89.3

Figure S12: ^{71}Ga MAS NMR spectrum of hot-pressed Ga-LLZO powder sample prepared with 5% excess Li in precursors at 16.4 T with a MAS speed of 40 kHz (black curve) along with fitting. The golden curve indicates the peak A, and the blue curve indicates the peak B. The red curve indicates the overall fit and the grey curve indicates the difference between the fitted and observed data. The table shows the fitted ^{71}Ga MAS NMR parameters (isotropic chemical shifts δ_{iso}, quadrupolar coupling constant C_Q and quadrupolar asymmetry parameter η_Q).

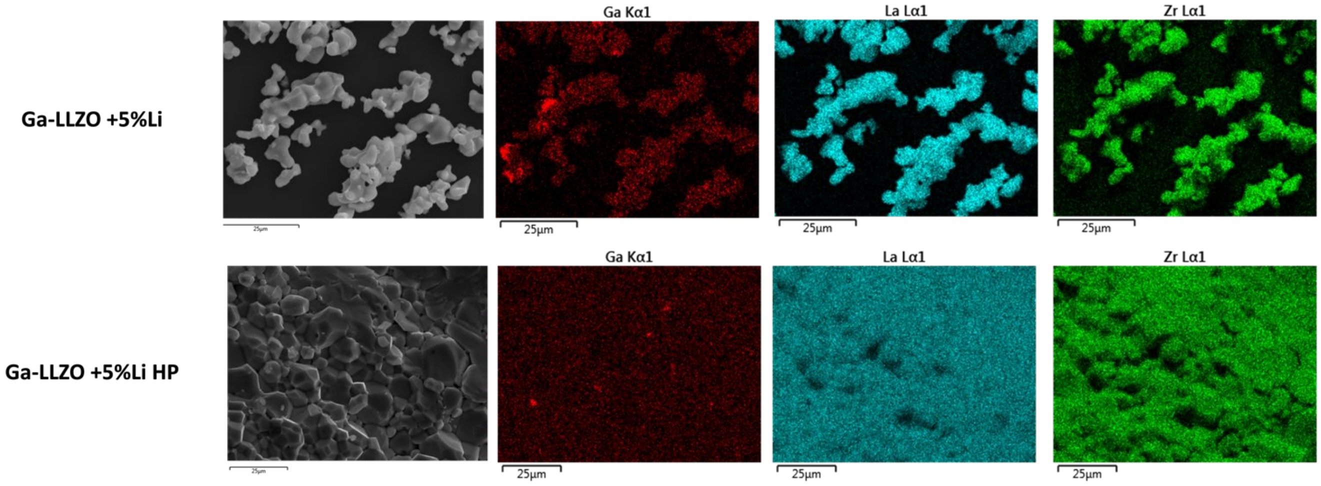

Figure S13: SEM-EDS maps Ga-LLZO powders prepared under different conditions

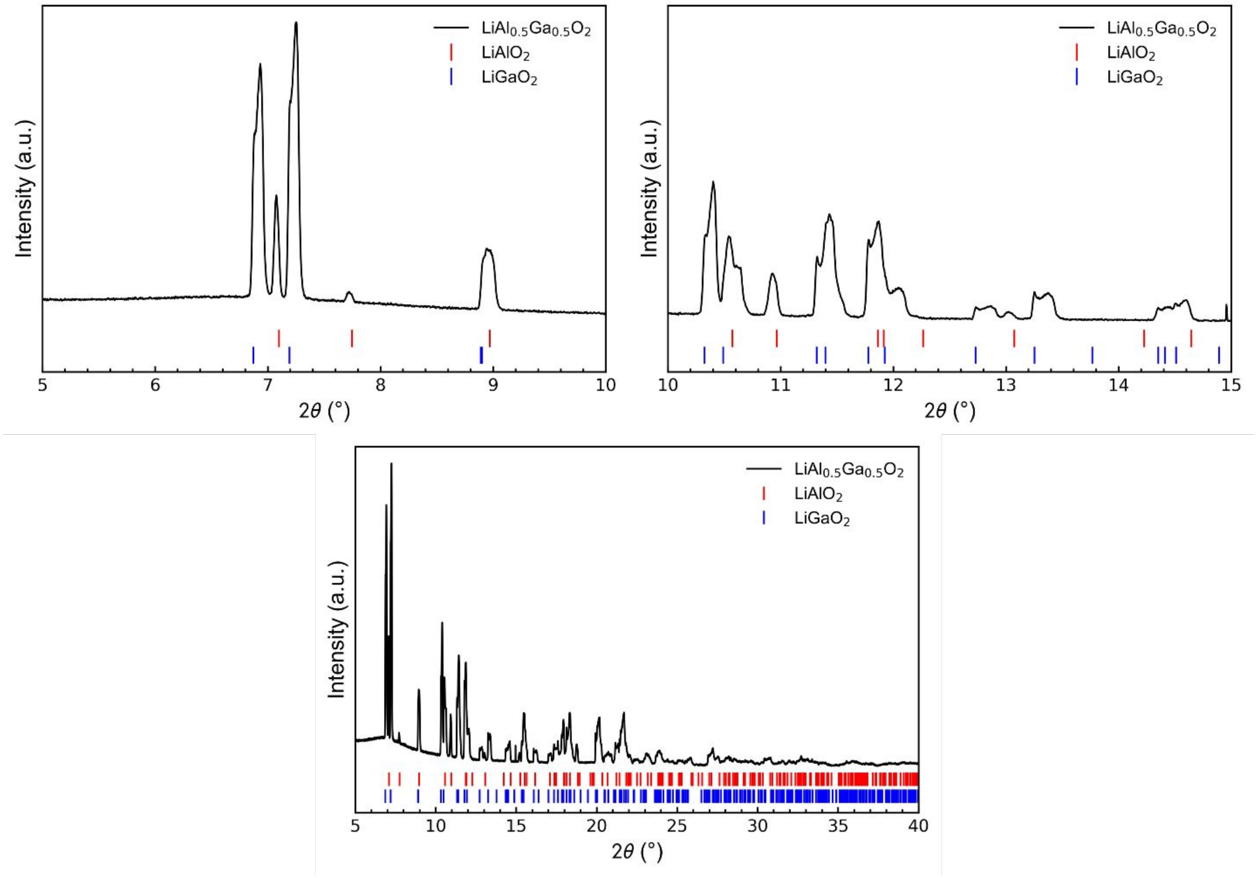

Figure S14: SXRD data of sample prepared by mixing precursors of $LiAlO_2$ and $LiGaO_2$ in 1:1 ratio and heating them (λ = 0.49381 Å). The red tick marks indicate the reflections from the $P4_12_12$ γ-$LiAlO_2$ phase. The dark blue tick marks indicate the reflections from $Pna2_1$ $LiGaO_2$ phase. The shifting of peaks to higher and lower 2θ than the peak positions corresponding to $LiGaO_2$ and $LiAlO_2$ and broadening of peaks at higher 2θ suggests cation disorder due to partial doping of these phases with Al and Ga respectively.

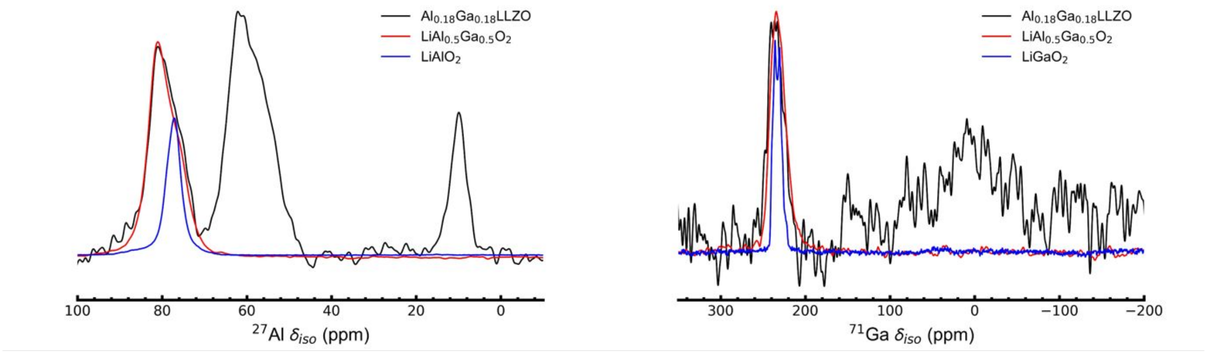

Figure S15: ^{27}Al and ^{71}Ga MAS NMR spectra of co-doped LLZO: $Li_{5.92}Al_{0.18}Ga_{0.18}La_3Zr_2O_{12}$ (overlayed with the MAS NMR spectrum of $LiAlO_2$/$LiGaO_2$ and $LiAl_{0.5}Ga_{0.5}O_2$). ^{27}Al MAS NMR spectra of co-doped LLZO and $LiAl_{0.5}Ga_{0.5}O_2$ were recorded at 30 kHz and ^{71}Ga MAS NMR spectra of co-doped LLZO and $LiAl_{0.5}Ga_{0.5}O_2$were recorded at 40 kHz (MAS NMR spectra of co-doped LLZO and $LiAl_{0.5}Ga_{0.5}O_2$ were recorded at 14.05 T, and $LiAlO_2$ and $LiGaO_2$ were recorded at 16.4 T and 40 kHz)
